# Genome-Wide Association Study of Peripheral Artery Disease

**DOI:** 10.1161/CIRCGEN.119.002862

**Published:** 2021-10-04

**Authors:** Natalie R. van Zuydam, Alexander Stiby, Moustafa Abdalla, Erin Austin, Emma H. Dahlström, Stela McLachlan, Efthymia Vlachopoulou, Emma Ahlqvist, Chen Di Liao, Niina Sandholm, Carol Forsblom, Anubha Mahajan, Neil R. Robertson, N. William Rayner, Eero Lindholm, Juha Sinisalo, Markus Perola, Milla Kallio, Emily Weiss, Jackie Price, Andrew Paterson, Barbara Klein, Veikko Salomaa, Colin N.A. Palmer, Per-Henrik Groop, Leif Groop, Mark I. McCarthy, Mariza de Andrade, Andrew P. Morris, Jemma C. Hopewell, Helen M. Colhoun, Iftikhar J. Kullo

**Affiliations:** Department of Immunology, Genetics and Pathology, Science for Life Laboratory, Uppsala University, Sweden (N.R.v.Z.).; Wellcome Centre for Human Genetics, Nuffield Department of Medicine (N.R.v.Z., M.A., A.M., N.R.R., N.W.R., M.I.M., A.P.M.), University of Oxford, United Kingdom.; Oxford Centre for Diabetes, Endocrinology and Metabolism, Radcliffe Department of Medicine (N.R.v.Z., A.M., N.R.R., N.W.R., M.I.M.), University of Oxford, United Kingdom.; Clinical Trial Service Unit and Epidemiological Studies Unit, Nuffield Department of Population Health (A.S., J.C.H.), University of Oxford, United Kingdom.; Department of Cardiovascular Medicine and the Gonda Vascular Center, Mayo Clinic, Rochester, MN (E. Austin, M.d.A., I.J.K.).; Folkhälsan Institute of Genetics, Folkhälsan Research Center, Helsinki, Finland (E.H.D., N.S., C.F., P.-H.G.).; Abdominal Center, Nephrology (E.H.D., N.S., C.F., P.-H.G.), University of Helsinki, Finland.; Heart and Lung Center (J.S.), University of Helsinki, Finland.; Vascular Surgery, Abdominal Center (M.K.), University of Helsinki, Finland.; Helsinki University Hospital, Research Program for Clinical and Molecular Metabolism, Faculty of Medicine (E.H.D., N.S., C.F., P.-H.G.), University of Helsinki, Finland.; Department of Medicine, Helsinki University Central Hospital (E.V.), University of Helsinki, Finland.; Institute for Molecular Medicine Finland (FIMM) (M.P., L.G.), University of Helsinki, Finland.; Usher Institute of Population Health Sciences and Informatics, The University of Edinburgh, United Kingdom (S.M., E.W., J.P.).; Genomics, Diabetes and Endocrinology, Lund University Diabetes Centre, Malmö, Sweden (E. Ahlqvist, E.L., L.G.).; Dalla Lana School of Public Health, University of Toronto, ON, Canada (C.D.L., A.P.).; Genetics & Genome Biology, SickKids, Toronto, ON, Canada (C.D.L., A.P.).; Department of Human Genetics, Wellcome Trust Sanger Institute, Hinxton, Cambridgeshire, United Kingdom (N.W.R.).; Finnish Institute for Health and Welfare, Helsinki, Finland (M.P., V.S.).; Ocular Epidemiology Research Group, University of Wisconsin-Madison (B.K.).; Pat Macpherson Centre for Pharmacogenetics and Pharmacogenomics, Ninewells Hospital and Medical School, University of Dundee, United Kingdom (C.N.A.P.).; Department of Medicine, Central Clinical School, Monash University, Melbourne, Victoria, Australia (P.-H.G.).; Oxford NIHR Biomedical Research Centre, Oxford University Hospitals Trust, United Kingdom (M.I.M.).; Now with Genentech, South San Francisco, CA (A.M., M.I.M.).; Department of Biostatistics, University of Liverpool, United Kingdom (A.P.M.).; Centre for Genetics and Genomics Versus Arthritis, Centre for Musculoskeletal Research, The University of Manchester, United Kingdom (A.P.M.).; Institute of Genetics and Molecular Medicine, University of Edinburgh, Western General Hospital Campus, United Kingdom (H.M.C.).

**Keywords:** diabetes, genome-wide association study, peripheral vascular disease, smoking

## Abstract

**Background:**

Peripheral artery disease (PAD) affects >200 million people worldwide and is associated with high mortality and morbidity. We sought to identify genomic variants associated with PAD overall and in the contexts of diabetes and smoking status.

**Methods:**

We identified genetic variants associated with PAD and then meta-analyzed with published summary statistics from the Million Veterans Program and UK Biobank to replicate their findings. Next, we ran stratified genome-wide association analysis in ever smokers, never smokers, individuals with diabetes, and individuals with no history of diabetes and corresponding interaction analyses, to identify variants that modify the risk of PAD by diabetic or smoking status.

**Results:**

We identified 5 genome-wide significant (*P*_*association*_ ≤5×10^−8^) associations with PAD in 449 548 (N_cases_=12 086) individuals of European ancestry near *LPA (lipoprotein [a]), CDKN2BAS1 (CDKN2B antisense RNA 1), SH2B3 (SH2B adaptor protein 3) - PTPN11 (protein tyrosine phosphatase non-receptor type 11), HDAC9 (histone deacetylase 9), and CHRNA3 (cholinergic receptor nicotinic alpha 3 subunit*) loci (which overlapped previously reported associations). Meta-analysis with variants previously associated with PAD showed that 18 of 19 published variants remained genome-wide significant. In individuals with diabetes, rs116405693 at the *CCSER1 (coiled-coil serine rich protein 1*) locus was associated with PAD (odds ratio [95% CI], 1.51 [1.32–1.74], *P*_*diabetes*_=2.5×10^−9^, *P*_*interactionwithdiabetes*_=5.3×10^−7^). Furthermore, in smokers, rs12910984 at the *CHRNA3* locus was associated with PAD (odds ratio [95% CI], 1.15 [1.11–1.19], *P*_*smokers*_=9.3×10^−10^, *P*_*interactionwithsmoking*_=3.9×10^−5^).

**Conclusions:**

Our analyses confirm the published genetic associations with PAD and identify novel variants that may influence susceptibility to PAD in the context of diabetes or smoking status.

## Abstract 2

Supplemental Digital Content is available in the text.

Peripheral artery disease (PAD) is a morbid form of atherosclerotic vascular disease that affects the lower limbs of >200 million people worldwide.^1^ PAD poses a significant health care burden with an estimated $21 billion spent annually on hospitalizations in the United States alone.^2^ Despite high mortality and economic impact, patients with PAD are underdiagnosed and undertreated.^3^

PAD is often classified as proximal and distal, subtypes that are associated with different risk factors and comorbidity profiles; type 2 diabetes (T2D) being more strongly associated with distal disease and smoking more strongly associated with proximal disease.^4^ Much remains unknown about the biology of PAD in individuals with diabetes,^5^ although proatherogenic changes, including chronic inflammation and hyperglycemia, are thought to increase the risk of PAD. Cigarette smoking increases the risk of all forms of atherosclerosis but is more strongly associated with PAD than with any other form of atherosclerotic cardiovascular disease.^6^

Genetic studies have been useful for elucidating pathways and factors that contribute to the development of other complex traits such as coronary artery disease (CAD) and T2D.^7,8^ These studies have also been successful in identifying context-specific variant effects, such as sex-specific effects.^9^ Fewer genetic association studies of PAD have been reported in contrast to other vascular traits, such as CAD.^10^ A recent study from the Million Veterans Program (MVP) and replication in the UK Biobank (UKBB) identified 19 loci associated with PAD in 31 307 PAD cases and 211 753 controls of mixed ancestry.^11^ In Individuals of East Asian descent, 3 variants—near *IPO5 (importin 5)/RAP2A (member of RAS oncogene family), EDNRA (endothelin receptor type A), and HDAC9 (histone deacetylase 9*)—have been reported at genome-wide significance.^12^ However, sample sizes remain considerably smaller than for other cardiovascular diseases, and previous studies have not assessed the relevance of phenotypic heterogeneity by examining the allelic effects by smoking or diabetes status genome wide.

In this study, we combined 11 independent genome-wide association studies (GWAS) of individuals of European ancestry (N_cases_=12 086 and N_controls_=449 548) to identify which genetic variants associated with PAD (primary analysis) and to assess whether there were any specific effects in individuals with smoking or diabetes in a stratified analysis. We performed 3 analyses: (1) A primary GWAS in all individuals to identify loci that contribute to PAD overall, irrespective of diabetes or smoking status; (2) GWAS analyses of PAD stratified by diabetes or smoking status to identify variants with smoking- or diabetes-specific effects; and (3) genome-wide interaction analyses of PAD stratified by diabetes status or smoking status to identify variants that interacted with either smoking or diabetes status to modify the risk of PAD. In addition, we attempted to assess whether PAD risk factors were linked to the development of PAD by performing genetic correlation analysis and contrasted these with associations with CAD.

## Methods

**Figure 1.**
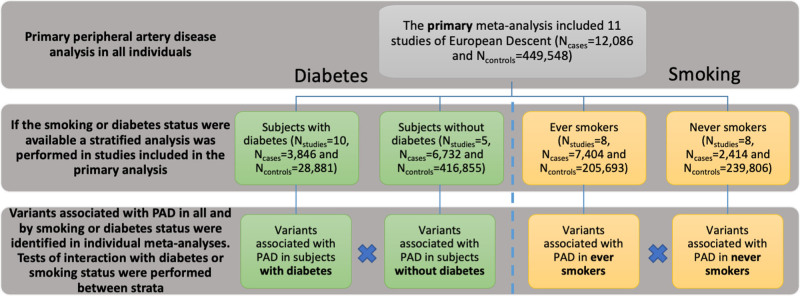
**Study design.** A primary meta-analysis of allelic effect on peripheral artery disease (PAD) in all individuals was performed. Subsequently and where smoking or diabetes status were available, individual study centers stratified by diabetes or smoking status and four additional meta-analyses of allelic effects on PAD were performed in: individuals with diabetes; individuals without diabetes; ever smokers and never smokers.

Summary level data from this study have been made publicly available via figshare (10.6084/m9.figshare.7811639). This study made use of data generated from individual studies for which the relevant institutional review board approval had been obtained and all participants consented to inclusion in individual studies. An overview of the study design is illustrated in Figure 1, and the methods are provided in the Data Supplement.

## Results

### Identification of Studies and Individuals to Include in the Meta-Analysis

The majority of PAD cases (N_cases_=7172) were identified using clinical parameters (eg, ankle-brachial index, a clinical diagnosis, procedures specific to PAD and treatment for claudication). A comparatively smaller subset of the PAD cases was identified based on a mixture of self-reported PAD in patients with clinical evidence of vascular disease and hospital admissions codes related to PAD (N_cases_=4914; Table I in the Data Supplement).

Most of our controls were individuals without any known history of vascular disease at the time of recruitment (N_controls_=419 548), in addition population controls who had population prevalence of vascular disease (N_controls_=2757, 0.6% of the total number of controls) and controls which may have had other types of vascular disease (N_controls_=27 102), were included. Any potential misclassification of controls is expected to be minimal (given a 5% population prevalence) and would contribute to more conservative results.

### Heritability

Heritability is the variability in a trait that can be explained by additive genetic variation. We were interested in whether the genetic heritability (in this case the chip heritability) was comparable to the heritability estimated in other studies.^13^ We used a population prevalence of 5% (which best matched the prevalence for the age range of samples included in this study)^14^ and found that the heritability for PAD was 55% (SE=9%) which was comparable to the narrow sense heritability of 48% estimated from twin studies.^13^ The heritability estimates for the diabetes- and smoking-stratified analyses were not reliable due to sample size and are thus not reported here.

### Primary GWAS of PAD

**Figure 2.**
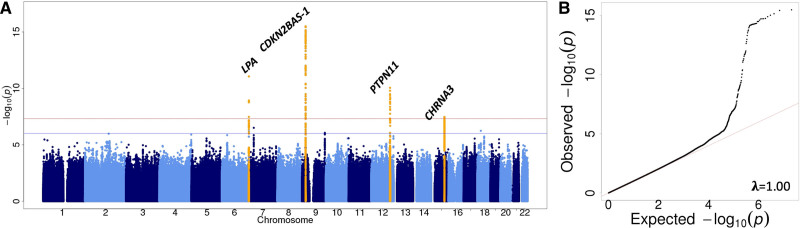
**Four loci were associated with peripheral artery disease overall.** A, A Manhattan plot shows genome-wide associations from the GoLEAD consortium at the *LPA (lipoprotein A), CDKN2BAS1 (CDKN2B antisense RNA 1), PTPN11 (protein tyrosine phosphatase, nonreceptor type 11), and CHRNA3 (cholinergic receptor nicotinic alpha 3 subunit*) loci with peripheral artery disease and (B) a QQ-plot shows a deviation from the normal distribution showing an inflation in genetic signal for variants associated with peripheral arterial disease in the GoLEAD consortium.

To identify variants associated with PAD, we combined summary statistics across GWAS in a fixed-effects meta-analysis, under an inverse-variance weighting scheme.^15^ Heterogeneity between studies was assessed using the Cochran *Q* test and *I*^2^. The primary genome-wide meta-analysis included 12 086 PAD cases and 449 548 controls with no known history of PAD from 11 studies of European ancestry (Figure 1; Tables I and II in the Data Supplement). We identified 4 loci, associated with PAD at genome-wide significance (*P*≤5×10^−8^), a threshold commonly used to declare association signals in GWAS (Figure 2A and 2B). We then conducted conditional analyses and identified a further independent signal at the *LPA* locus (rs7452960; Figure 2A, Table and Figure I in the Data Supplement). In summary, 2 independent index SNPs at the *LPA (lipoprotein [a]*) locus were identified (rs7452960 and rs10455872), which have also been associated with Lp(a) (lipoprotein [a]) levels^16,17^ and CAD previously; one index SNP at the *CDKN2BAS1 (CDKN2B antisense RNA 1*) locus (rs10738610) that has previously been associated with CAD and T2D^7,8^; another at the *SH2B3 (SH2B adaptor protein 3) - PTPN11 (protein tyrosine phosphatase non-receptor type 11*) locus (rs10774624) that has also been associated with type 1 diabetes and chronic kidney disease; and another at the *CHRNA3 (cholinergic receptor nicotinic alpha 3 subunit*) locus (rs1317286) that was also associated with smoking and lung cancer (Table III in the Data Supplement). These associations overlapped with associations reported at these loci by the MVP.^11^

**Table 1.**
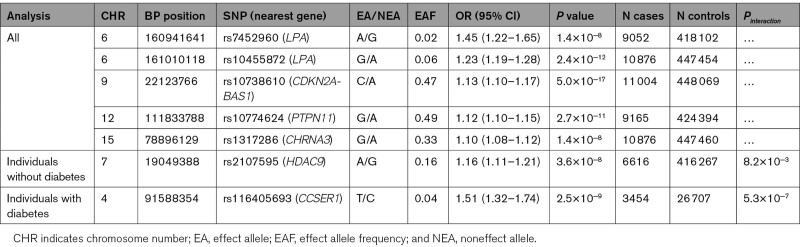
Seven Lead Variants Were Associated With Peripheral Artery Disease at Genome-Wide Significance (*P*≤5×10^−8^) in the Genetics of Lower Extremity Arterial Disease (GoLEAD) Consortium (Including the UK Biobank)

#### Replication of Previously Published Loci

There were 19 published variants for PAD in individuals of different ancestries reported from a previous meta-analysis of MVP+UKBB. Meta-analysis of the primary GWAS of PAD from GoLEAD (excluding UKBB) with published summary statistics from MVP+UKBB supported the association of 18 of the 19 published variants for the risk allele (Figure II and Table IV in the Data Supplement). The lead variant at *MMP3 (matrix metallopeptidase 3*) remained associated with PAD but above the genome-wide significance threshold (odds ratio [OR; 95% CI], 1.07 [1.04–1.09], *P*=2.6×10^−7^). These associations are supported by studies which used different definitions of cases and controls, for example, electronic health records and clinical diagnoses.

We were interested in whether variants associated with PAD in populations of East Asian ancestry (N_cases/controls_=3164/20 134)^12^ were also associated with PAD in populations of European ancestry. These may highlight common pathways contributing to PAD across these populations. The variants associated with PAD in individuals of East Asian ancestry (rs2074633, rs9584669, and rs6842241) showed varying associations with PAD in individuals of European ancestry (Table V in the Data Supplement). We found rs2074633 (effect allele frequency [EAF]_EA_=0.38), near *HDAC9*, to be associated with PAD (EAF_Euro_ 0.22; OR [95% CI], 1.09 [1.05–1.12]; *P*_*association*_=6.0×10^−6^), albeit not at genome-wide significance, when comparing this to the East Asian population (EAF_EA_ 0.38; OR [95% CI], 1.16 [1.10–1.22]; *P*_*association*_=8.43×10^−8^) the OR is directionally consistent. *HDAC9* is a locus that has been reported for PAD across different ancestries. Rs9584669 (EAF_EA_=0.94), near *IPO5*, was not associated with PAD in our cohort (EAF_Euro_ 0.87; OR [95% CI], 1.02 [0.97–1.06]; *P*_*association*_=0.48). Rs6842241 (EAF_EA_=0.70), near *EDNRA*, showed modest association, but in the opposite direction, with PAD (EAF_*Euro*_ 0.86; OR [95% CI], 0.94 [0.90–0.98]; *P*_*association*_=3.4×10^−3^). The allele frequencies for rs6842241 were similar in individuals of East Asian and European ancestry. The inverse association in individuals of European ancestry could be due to chance as it did not surpass genome-wide significance or could reflect differences in linkage disequilibrium or risk factors between the 2 populations.

### Diabetes- and Smoking-Stratified GWAS Meta-Analyses

Identification of genetic factors that have stratum specific associations may indicate that different pathways are important to the development of PAD based on risk factor context. To identify these factors, we performed diabetes- and smoking-stratified analyses in the samples used for the primary PAD analysis (Figure 1). The PAD cases and PAD free controls were stratified by smoking status in 8 of the 11 studies (7404 PAD cases and 205 693 PAD free controls among ever smokers; and 2414 PAD cases and 239 806 PAD free controls among never smokers) and by diabetes status in 10 of the 11 studies (3846 PAD cases and 28 881 PAD free controls with diabetes; and 6732 PAD cases and 416 855 PAD free controls without diabetes; Table II in the Data Supplement).

First, we identified variants that were associated with PAD at genome-wide significance (*P*≤5×10^−8^) in individual strata: ever smokers; never smokers; individuals with diabetes; and individuals with no history of diabetes. We then performed genome-wide interaction analyses with smoking or diabetes status respectively (Data Supplement) to identify genetic variants that interacted with the risk factor to modify the risk of PAD. These analyses used the stratified GWAS to test for interaction by comparing the differences in allelic effects between strata for all SNPs rather than combining allelic interaction effects from individual studies.

### Diabetes Stratified Association Analysis

Rs116405693, a novel index variant near *CCSER1 (coiled-coil serine rich protein 1*), was associated with PAD in individuals with diabetes (EAF_*diabetes*_, 0.04, OR_*diabetes*_ [95% CI], 1.51 [1.32–1.74), *P*_*diabetes*_=2.5×10^−9^; Table and Figure III in the Data Supplement); but not in individuals without diabetes despite power to detect an effect (OR [95% CI], 0.97 [0.87–1.08], *P*_*nodiabetes*_=0.58, *P*_*interactionwithdiabetes*_=5.3×10^−7^; power to detect in individuals without diabetes ≥80%, α=5.0×10^−8^; Figure III in the Data Supplement). The variant showed strong evidence of interaction with diabetes status and is likely to represent a diabetes specific effect, suggesting that different pathways may play a role in the development of PAD in individuals with diabetes compared with individuals without diabetes. No other variants reached genome-wide significance in this analysis.

The MVP reported an interaction for rs3104154 with T2D to modify the risk of PAD (*P*_*interaction*_=3.0×10^−8^), this was on the relative risk scale from a study which considered only variants also associated with T2D.^18^ In this study, using exponential odds, we found no evidence for interaction of rs3104154 with diabetes status (EAF, 0.95, OR_diabetes_ [95% CI], 0.95 [0.90–1.23], *P*_*diabetes*_=0.54, OR_nodiabetes_ [95% CI], 0.94 [0.94–1.19], *P*_nodiabetes_=0.38, *P*_interactionwithdiabetes_>0.99). The lack of replication could be due to the different scales used.

In individuals without diabetes, 3 index variants were associated with PAD at genome-wide significance. Two of these associations were the same index SNPs or proxies thereof reported in the primary PAD analysis near *CDKN2BAS-1* and *CHRNA3.* Rs2107595, an index SNP near *HDAC9*, was associated with PAD in individuals without diabetes (EAF_*nodiabetes*_ 0.16; OR_*nodiabetes*_ [95% CI], 1.16 [1.11–1.21], *P*_*nodiabetes*_=3.6×10^−8^) but was not detected in the primary PAD analysis or in individuals with diabetes (Table, Figure 1, Table VI and Figure IV in the Data Supplement). However, this is not a stratum specific effect, the combined allelic effects from GoLEAD+UKBB+MVP for rs2107595 showed that this variant was associated with PAD overall (*P*_*association*_=4.2×10^−11^; Table IV in the Data Supplement). We could not find any evidence to support differing pathways contributing to the development of PAD in subjects without diabetes compared with the primary analysis.

### Smoking Stratified Association Analyses

The same lead variants or their proxies at the *LPA*, *CDKN2BAS-1*, and *CHRNA3* loci that were reported in the primary PAD GWAS were associated with PAD in ever smokers. However, the lead variant, rs12910984 (EAF 0.76, OR_smokers_ [95% CI], 1.15 [1.11–1.19], *P*_*smokers*_=9.3×10^−10^), at the *CHRNA3* locus, showed evidence of interaction with smoking status (*P*_*interactionwithsmoking*_=3.9×10^−5^; power to detect an OR=1.15 for EAF=0.76 in never smokers ≥80%; Figure V, Tables VII and VIII in the Data Supplement). The *CHRNA3* locus was also associated with PAD in the overall cohort. *CHRNA3* is a known risk factor for smoking, nicotine dependence, and greater smoking quantity.^19^ In the stratified analysis, this locus was associated in smokers but not in nonsmokers. This may suggest that the association in the general population may be partly driven by those who smoke. A novel association for index variant rs200841208, in *HLA-DRB2 (Major histocompatibility complex, class II, DR beta 2 [pseudogene]*), was detected for PAD in ever smokers (OR [95% CI], 1.35 [1.18–1.55], *P*_*smokers*_=3.6×10^−8^, *P*_*interactionwithsmoking*_=2.3×10^−4^), but not in never smokers (OR [95% CI], 0.95 [0.81–1.11], *P*_*neversmokers*_=0.51; Figure V and Table VII in the Data Supplement). This region is well known for its complex linkage disequilibrium and our finding will require replication in an independent sample.

#### Post Hoc Power and Sample Size Calculations to Detect Interactive Effects

A substantial challenge in detecting loci that interact with diabetes or smoking status to modify the risk of PAD is sufficient sample size. The significance of an interaction is determined by the size of the difference in allelic effects between strata and how well those allelic effects are estimated in each stratum (SE, a function of sample size). Power analyses were based on the following sample sizes: smokers (N_cases_=7404 and N_controls_=205 693) versus nonsmokers (N_cases_=2414 and N_controls_=239 806); and diabetes (N_cases_=3846 and N_controls_=28 881) versus no diabetes (N_cases_=6732 and N_controls_=416 855).

We had ≥80% power, at either α=5×10^−4^ (Bonferroni correction for 100 SNPs selected for replication) or α=5×10^−8^, to detect large differences (15%–40%) in allelic effects between strata for SNPs with EAF>0.1 where the allelic effects were either in opposite directions (ie, OR_nonsmoker_=0.80 and OR_smoker_=1.20), or there was no effect in one stratum and an effect in the other stratum (ie, OR_nonsmoker_=1.00 and OR_smoker_=1.15; Figure VI and Table VIII in the Data Supplement). There was <80% power to detect interactions where the allelic effects in each stratum were in the same direction (ie, OR_nonsmoker_=1.10 and OR_smoker_=1.30). To replicate the interaction findings from this study, we would need a similar number of cases in each stratum included in this study (Table IX and Methods in the Data Supplement).

### Shared Genetic Background With Other Traits

PAD and CAD are often comorbid and share many common risk factors. To understand whether these risk factors—represented by their underlying genetic variation—may affect the risk of PAD and CAD differently, we performed genetic correlation for 6 common risk factors. Pairwise genetic correlation analyses were performed for PAD and CAD separately with body mass index,^20^ HDL-C (high-density lipoprotein cholesterol), LDL-C (low-density lipoprotein cholesterol), triglycerides,^21^ T2D,^22^ systolic blood pressure (UKBB automated reading). Positive correlations indicate that genetic variation associated with higher levels of the trait are associated with higher risk of PAD (or CAD), whilst a negative correlation indicates that the genetic variation associated with higher levels of the trait is associated with lower risk of PAD (or CAD). As anticipated, there was strong genetic correlation for PAD with CAD (rg 0.58 [95% CI, 0.44–0.71], *P*=1.1×10^−16^; Table X in the Data Supplement). The pattern of genetic correlation of PAD and CAD with 6 risk factors was broadly similar across PAD and CAD with similar estimates of genetic correlation for both diseases. Body mass index, T2D, LDL-C, triglycerides, and blood pressure were positively correlated with both PAD and CAD while HDL-C was negatively correlated with PAD and CAD (Table X in the Data Supplement). The genetic correlation estimates are affected by the heritability of the traits being compared, a low heritability in one of the traits can result in a weak genetic correlation. These results are in line with the epidemiological associations for these traits.

## Discussion

We identified 5 genetic variants associated with PAD at genome-wide significance in our study and one variant associated with PAD in those with diabetes. Additionally, we found a suggestive association for a variant at the *HLA-DRB2* locus with PAD in ever smokers. Our analysis supported the association of 18 of the 19 published genetic associations for PAD in the largest sample for PAD reported to date. The study by the MVP relied on PAD identified through electronic health records but, in this study, we validate the previous associations in PAD defined using multiple and different sources. Genetic correlation analyses suggest major similarities in common risk factors between PAD and CAD, in line with their shared atherosclerotic mechanism.

Many of the loci associated with PAD are also known CAD loci, that is, *LPA*, *CDKN2BAS-1, HDAC9*, and *SH2B3/PTPN11*. *CDKN2BAS-1* and *HDAC9* have also been associated with CAD and large artery stroke.^8,23^ While the overlap of genetic determinants is unsurprising due to the shared underlying atherosclerotic processes, they are not identical in terms of genetic risk. The *PTPN11* locus has also been associated with CAD, but the index variant associated with PAD also overlapped associations with lower glomerular filtration rate and higher blood pressure. Chronic kidney disease is correlated with higher risk of PAD suggesting an overlap in pathways contributing to hypertension, chronic kidney disease, and PAD.^24^ The genetic associations in this study indicate that the biological factors contributing to the development of PAD are not identical to the development of CAD. There are differences in the genetic determinants by smoking and diabetes status which are not observed in large stratified GWAS analyses of CAD.^25–27^ The *CCSER1* locus showed effects on PAD specific to the context of diabetes but not much is known about this locus and it requires further investigation. These genetic differences indicate that there may be pathways that could be targeted for therapeutic development that would be distinct from therapeutics for CAD.

Smoking status and smoking quantity are the strongest risk factors for PAD in the general population.^6^ In prior GWAS, the lead variant at the *CHRNA3* locus overlapped with variants that were also associated with predisposition to become a smoker, smoking quantity, lung cancer, and chronic obstructive pulmonary disease.^28^ The same variant showed interaction with smoking status reflecting the association of the locus with predisposition to smoking and that the association with increased smoking quantity is an important risk factor for PAD in smokers. We also detected an association near *HLA-DRB1* with PAD in ever smokers only. However, the lead variant was not well imputed in larger cohorts (imputation information=0.61) and absent from many of the smaller studies. It is also in a region of the genome that has complex linkage disequilibrium structures; thus, it would be necessary to confirm this association in independent samples. Our results indicate that there are likely to be different biological mechanisms contributing to the development of PAD in patients with diabetes and in smokers, potentially reflecting the phenotypic differences between distal and proximal PAD. This supports the clinical data which shows different manifestations of PAD dependent on risk factor context.^4^

The *LPA* variants associated with PAD in our study, irrespective of smoking or diabetes status have also been associated with plasma Lp(a) levels.^16,17^ Mendelian randomization studies in the UKBB have also shown that one SD reduction in Lp(a) levels was associated with a 31% lower risk of PAD. Therefore, genetic evidence suggests that new treatments currently under development that lower serum Lp(a) levels have the potential to lower the risk of PAD.^29,30^ A phase 3 randomized, double blinded, placebo-controlled trial is currently evaluating the effect on Lp(a) lowering by antisense approach (AKCEA-APO(a)-L_Rx_), on cardiovascular outcomes.^30–32^

The main limitation of this study is a lack of independent replication for the main GWAS and for the stratified analyses. As we have demonstrated, through replication of GWAS hits previously reported in the MVP+UKBB, further studies can help to provide such support. Future meta-analyses will include additional samples and the opportunity to confirm the loci, we have identified both within those of European ancestry as well as to undertake transethnic meta-analyses that will allow exploration of the overlap of these loci across ancestries.

Our results demonstrate that context dependent genetic factors are operative in PAD and highlight the importance of analyses stratified by diabetes and smoking—the main risk factors for PAD. These results are also consistent with clinical observations of 2 subtypes of PAD, proximal disease strongly associated with smoking and distal, disease strongly associated with diabetes.^4^ Future work should focus on mechanistic studies to investigate how genetic variation at these loci influence pathophysiologic processes relevant to PAD will aid in our understanding of the molecular genetic basis of PAD and development of new therapeutic targets.

## Acknowledgments

We acknowledge the contribution of summary statistics data from CARDIoGRAMplusC4D, the UK Biobank, the MAGIC consortium, ISGC, GIANT, the EGG consortium, TAG, and DIAGRAM. Part of this work was conducted using the UK Biobank resource under application number 9161. The analysis of individual studies was performed by Dr van Zuydam, Dr Stiby, Dr Austin, Dr Dahlström, Dr McLachlan, Dr Vlachopoulou, Dr Ahlqvist, C. Di Liao, Dr Sandholm, Dr Mahajan, N.R. Robertson, Dr Sinisalo, Dr Perola, Dr Weiss, Dr Price, Dr Paterson, Dr de Andrade, Dr Hopewell, and Dr Kullo. Central data analysis was performed by Dr van Zuydam, Dr Stiby, M. Abdalla, Dr Dahlström, Dr McCarthy, and Dr Morris. Individual study design was performed by Dr Dahlström, Dr Sandholm, Dr Lindholm, Dr Sinisalo, Dr Kallio, Dr Price, Dr Klein, R.K., Dr Salomaa, Dr Palmer, Dr Groop, Dr Groop, Dr de Andrade, Dr Hopewell, Dr Colhoun, and Dr Kullo. Article preparation done by Dr van Zuydam, Dr Stiby, M. Abdalla, Dr Austin, Dr Dahlström, Dr McLachlan, Dr Vlachopoulou, Dr Ahlqvist, C. Di Liao, Dr Sandholm, Dr Forsblom, Dr Mahajan, N.R. Robertson, Dr Rayner, Dr Lindholm, Dr Sinisalo, Dr Perola, Dr Kallio, Dr Weiss, Dr Price, Dr Paterson, Dr B Klein, Dr R Klein, Dr Salomaa, Dr Palmer, Dr Groop, Dr Groop, Dr McCarthy, Dr de Andrade, Dr Morris, Dr Hopewell, Dr Colhoun, and Dr Kullo. Sample collection done by Dr Dahlström, Dr Forsblom, Dr Lindholm, Dr Sinisalo, Dr Perola, Dr Kallio, Dr Price, Dr B Klein, Dr R Klein, Dr Salomaa, Dr Palmer, Dr Groop, Dr Groop, Dr Hopewell, and Dr Kullo. Approval of final article was done by all authors.

## Sources of Funding

This study was supported by: European Union’s Seventh Framework Program (FP7/2007–2013) for the Innovative Medicine Initiative under grant agreement IMI/115006 (the SUMMIT consortium); the Aarno Koskelo Foundation; the Academy of Finland (299200, 316664); Professor Kullo is additionally supported by the National Heart, Lung, and Blood Institute (NHLBI) grant K24-HL707710; Dr Hopewell acknowledges personal support from the British Heart Foundation (FS/14/55/30806); the Chief Scientist Office of Scotland (Project Grant CZB/4/672); DOLOrisk (European Union’s Horizon 2020 research and innovation programme grant No 633491); the European Foundation for the Study of Diabetes (EFSD); EFSD/Sanofi European Diabetes Research Programme in Macrovascular Complications; the Finnish Diabetes Research Foundation; the Finnish Foundation for Cardiovascular Research; the Folkhälsan Research Foundation; the Helsinki University Central Hospital special government funds (nos. TYH2012209, TYH2014312, and TYH2017250); the Juvenile Diabetes Research Foundation (2-SRA-2014-276-Q-R); the Liv och Hälsa Foundation; the Mayo Foundation; the National Human Genome Research Institute (NHGRI, HG04599, and HG06379); the National Institute of General Medical Sciences (NIGMS), Bethesda, MA; NIH: U01-DK105535; Nylands Nation; Oxford University; TRIiC (the Novo Nordisk Foundation); the UK Medical Research Council (MRC); the Waldemar von Frenckell Foundation; the Wellcome Trust (076113, 090532, 098381, 203141, and 212259); and the Wilhelm and Else Stockmann Foundation.

## Disclosures

Dr Groop is an advisory board member for AbbVie, AstraZeneca, Boehringer Ingelheim, Janssen, Medscape, MSD, Novartis, Novo Nordisk, and Sanofi. Dr Colhoun receives research support and honorarium and is also a member of the advisory panels and speaker’s bureaus for Sanofi Aventis, Regeneron, and Eli Lilly. Dr Colhoun has been a member of the Data and Safety Monitoring Board of the Advisory Panel for the CANTOS trial (Canakinumab Anti-Inflammatory Thrombosis Outcomes Study; Novartis Pharmaceuticals). Dr Colhoun also receives or has recently received a nonbinding research support from Roche Pharmaceuticals, Pfizer Inc, Boehringer Ingelheim, and AstraZeneca LP. Dr Colhoun is a shareholder of Roche Pharmaceuticals and Bayer. All of the above is outside the submitted work. Dr Salomaa has received honoraria from Novo Nordisk and Sanofi for consulting, unrelated to the present work. He also has ongoing research collaboration with Bayer, outside the present work. The views expressed in this article are those of the author(s) and not necessarily those of the National Health Service, the NIHR, or the Department of Health. Dr McCarthy was a Wellcome Investigator and an National Institute for Health Research Senior Investigator, has served on advisory panels for Pfizer, NovoNordisk and Zoe Global, has received honoraria from Merck, Pfizer, Novo Nordisk and Eli Lilly, and research funding from Abbvie, AstraZeneca, Boehringer Ingelheim, Eli Lilly, Janssen, Merck, NovoNordisk, Pfizer, Roche, Sanofi Aventis, Servier, and Takeda. As of June 2019, Drs Mahajan and McCarthy are employees of Genentech, and holders of Roche stock. As of September 2019, Dr van Zuydam is an employee of AstraZeneca PLC and a holder of AstraZeneca stock. As of 2016, Dr Vlachopoulou is an employee of Medpace. As of November 2016, C. Di Liao is an employee of Roche Pharmaceuticals and is a holder of Roche stock.

## Supplemental Materials

Supplemental Methods

Supplemental Tables I–XII

Supplemental Figures I–VI

References ^33–48^

## Supplementary Material



## Appendix

GoLEAD: Barbara Klein, Veikko Salomaa, Emma Ahlqvist, Leif Groop, Eero Lindholm, Per-Henrik Groop, Emma Dahlström, Carol Forsblom, Niina Sandholm, Andrew Paterson, Colin NA Palmer, Helen M Colhoun, Natalie R van Zuydam, Jemma C Hopewell, Alexander Stiby, Sólveig Grétarsdóttir, Guðmar Þorleifsson, Unnur þorsteinsdóttir, Kari Stefansson, Iftikhar Kullo, Mariza de Andrade, Mark I McCarthy, Andrew P Morris and Jackie Price

SUMMIT: Michael Mark, Timo Kanninen, Barbara Thorand, Giuseppe Remuzzi, David Dunger, Angela Shore, Ulf Smith, Per-Henrik Groop, Veikko Salomaa, Seppo Ylä-Herttuala, Claudio Cobelli, Riccardo Bellazzi, Ele Ferrannini, Carlo Patrono, Pirjo Nuutila, Paul McKeague, Birgit Steckel-Hamann, Li-ming Gan, Everson Nogoceke, Piero Tortoli, Bernd Jablonka, Mary-Julia Brosnan.
