# Supplementary material for: Genome-Wide Association Study of Peripheral Artery Disease
Source: Circ Genom Precis Med. 2021 Oct 4;14(5):e002862. doi: 10.1161/CIRCGEN.119.002862 (PMC8542067; doi:10.1161/CIRCGEN.119.002862)
Supplement: Supplementary file 1 [file hcg-14-e002862-s001.pdf]

SUPPLEMENTAL MATERIAL

Table of Contents

Supplemental Methods 3
Phenotype description by study 3
Corogene 3
Diabetes Control and Complications Trial (DCCT)/ Epidemiology of Diabetes Interventions and Complications (EDIC) 4
deCODE 5
Edinburgh artery study 5
The Finnish Diabetic Nephropathy Study 6
Mayo Clinic 9
Scannia Diabetes Registry 10
The UK Biobank 11
The Wisconsin Epidemiologic Study of Diabetic Retinopathy: XVII 12
Genome-wide association studies 12
Meta-analysis 13
Estimation of heritability 13
Conditional analysis 14
PhenoScanner 14
Post hoc power calculations for individual SNP associations 14
Interaction analysis post hoc power calculation 15
Genetic correlation analyses 16
Supplemental Tables 17
Supplemental Table I 17
Supplemental Table II 25
Supplemental Table III 26
Supplemental Table IV 30
Supplemental Table V 32
Supplemental Table VI 35
Supplemental table VII 36
Supplemental Table VIII 37
Supplemental table IX 38
Supplemental Table X 39

Supplemental Table XI 40
Supplemental Table XII 49
Supplemental Figures 50
Supplemental Figure I 51
Supplemental Figure II 52
Supplemental Figure III 53
Supplemental Figure IV 54
Supplemental Figure V 55
Supplemental Figure VI 56

Supplemental Methods

Phenotype definitions and ankle brachial index (ABI) measurements, characteristics/procedures, electronic health care records, medications. A description of the PAD definition employed by each study is given in Supplemental Table I. Individuals with type 1 diabetes (T1D) or T2D were identified based on a clinical diagnosis or self-report. Ever smokers were defined as individuals with a record of smoking at present and/or in the past and never smokers were defined as individuals with no recorded history of smoking. The individual studies included in this meta-analysis were approved by the appropriate institutional review board and/or that appropriate informed consent was obtained from human subjects. Studies with relevant data to identify individuals with and without history of PAD, linked to high-density genotype data were considered for inclusion in these meta-analyses. Case definition varied slightly by study due to different healthcare data availability; all definitions were agreed by clinically trained members of the collaboration prior to inclusion (Supplemental Table I).

Phenotype description by study

Corogene

The Corogene study is a prospective cohort study where 5000 consecutive Finnish patients assigned to coronary angiogram in the region of Helsinki University Central Hospital are included. The goal was to study more than 5000 patients in the first cohort, which was achieved during a 20-month period (June 2006 to March 2008). A new cohort of 5000 patients will be collected every 5 years within a period of 12 months to study the trends in heart disease and coronary risk factors. The next cohort is scheduled to be collected in 2013.

The Hospital District of Helsinki and Uusimaa comprises a population of 1.5 million, and includes four hospitals with coronary angiogram laboratories, three of which (Helsinki University Central Hospital, Jorvi Hospital and Peijas Hospital) are supervised by the Helsinki University Central Hospital. The fourth unit is in a private hospital. During the study period, over 4800 coronary angiograms were performed annually within the region. Helsinki University Central Hospital covers 75%, Jorvi Hospital

16%, Peijas Hospital 4% and the private hospital 5% of these angiograms. ST-elevation myocardial infarction patients are referred exclusively to Helsinki University Central Hospital. During off-duty hours, the region sending ST-elevation myocardial infarction patients for angiogram is wider; it includes about two million inhabitants.

Each patient filled a two-page questionnaire, which included anthropometry (height), medication, heart symptoms (which kind and for how long, rhythm problems), other diseases (hypertension, diabetes, hypercholesterolaemia, claudication, brain insults, myocardial infarctions) and heart diseases or sudden death of relatives (who, when, at what age and which disease). A computerized database was used for the collection of extensive information from hospital records. Trained nurses, supervised by physicians, collected the information from hospital documents (patient records and coronary-angiogram database). All relevant information on previous medical conditions, medication used, cardiovascular risk factors, laboratory measurements, coronary angiography, electrocardiogram (ECG), echocardiography, and thorax X-ray was collected.

Cases: Were defined as individuals with a clinical record of an ABI at rest or post exercise of <0.9 or surgical interventions related to PAD

Controls: Were free of any PAD

Diabetes Control and Complications Trial (DCCT)/ Epidemiology of Diabetes Interventions and Complications (EDIC)

The Diabetes Control and Complications Trial (DCCT) randomly assigned 1,441 patients with type 1 diabetes to intensive versus conventional therapy for a mean of 6.5 years, after which 93% were subsequently monitored during the observational Epidemiology of Diabetes Interventions and Complications (EDIC) study.

Cases: PAD was defined as a clinical record of an ankle brachial index at rest or post exercise of <0.9 and/or an ankle systolic blood pressure >255mmHg (excluding any non-atherosclerotic causes of PAD) and/or amputations from the thigh through to midfoot (excluding any non-vascular amputations).

Controls: Were identified as individuals with no history of PAD.

deCODE

For all studies involving Icelandic individuals, the study protocols were approved by the National Bioethics Committee and the Data Protection Authority (DPA) of Iceland. The DPA encrypted all personal identifiers associated with information or blood samples using the third-party encryption system[33]. Iceland Patients have been recruited over the past nine years, as part of a genetic study at deCODE.

Cases: PAD was defined as a clinical diagnosis of PAD at the major hospital in Reykjavik, the Landsite University Hospital, during the years 1983 to 2006 from registry data. Diagnosis was confirmed by vascular imaging or segmental pressure measurements.

Controls: Included individuals with no history of any vascular disease

Edinburgh artery study

The target population comprised the inhabitants of Edinburgh aged 55 to 74 years. An age-stratified random sample was selected from the age-sex registers of ten general practices with catchment populations spread geographically and socioeconomically throughout the city. Following ten minutes' rest in the supine position, systolic and diastolic (Phase V) blood pressures were taken in the right arm using a Hawksley random zero sphygmomanometer. The femoral, posterior tibial and dorsalis pedis arteries were palpated in both legs. Ankle systolic blood pressures in the right then left leg were taken using the random zero sphygmomanometer and a Sonic aid Doppler probe. Blood flow was detected where possible in the posterior tibial artery. In the reactive hyperaemia test which followed, ankle systolic pressure was measured in the right and left legs 15 seconds after the release of a cuff occluding arterial flow just above the knee for four minutes at about 50 mm Hg above systolic pressure. The timing was standardized using an electronic timer.

Cases: PAD was defined as intermittent claudication (IC), IC was diagnosed using the WHO questionnaire. Multiple events of the same type occurring in the same subject were reported only once.

Controls: Were defined as individuals with no history of PAD.

The Finnish Diabetic Nephropathy Study

The Finnish Diabetic Nephropathy (FinnDiane) Study is an ongoing prospective nationwide study designed to identify genetic, clinical, biochemical and environmental risk factors for diabetic complications with focus on diabetic nephropathy. Briefly, all patients went through a comprehensive clinical examination during which blood and urine samples were collected, and standardized questionnaires regarding health and medical history were completed[34]. The genome-wide genotyping of individuals was performed as previously described[35]. Further information on clinical events has been retrieved from the National Care Register of Health Care, which is the national hospital discharge register in Finland. In FinnDiane we defined cases and controls for PAD based on information retrieved from the hospital discharge register. Cases and controls were analysed cross-sectionally.

Cases: As cases we considered patients with a procedure code for peripheral artery bypass surgery or a lower percutaneous angioplasty for PAD or a major amputation. A vascular surgeon (M.K.) verified all cases by reviewing medical papers and removed patients with any non-vascular causes (such as cancer or trauma).

Controls: As controls we considered individuals not meeting the case criteria above, no record of amputations (neither major nor minor) and a diabetes duration exceeding 15 years.

The Genetics of Diabetes and Audit Research in Tayside Scotland (GoDARTS)

GoDARTS is a longitudinal case/control study of type 2 diabetes of ~16,000 individuals. Study participants are linked to electronic medical records and vascular laboratory data that were used to identify individuals with PAD.

Cases: were defined as having ankle brachial pressure index <0.9 or an ankle systolic pressure of >255mmHg excluding non-atherosclerotic causes of PAD; >2 occurrences of the following codes: ICD9: 747.22,237.7,443.1,446.0,446.4,446.5,446.6,446.7,447.6,710.1,747.1,747.64; ICD10: I73.1,I77.6,M30.3,M31.1,M31.30,M31.4,M31.6,M34.9,Q25.1,Q25.2,Q25.3,Q27.32,Q85.00 or local equivalent; or a hospital admissions code of PAD diagnosis: ICD9: 440.2×,440.3×, or 440.8×; *ICD10* excluding non-atherosclerotic causes of PAD; >2 occurrences of the following codes ICD9: 747.22,237.7,443.1,446.0,446.4,446.5,446.6,446.7,447.6,710.1,747.1,747.64; ICD10: I73.1,I77.6,M30.3,M31.1,M31.30,M31.4,M31.6,M34.9,Q25.1,Q25.2,Q25.3,Q27.32,Q85.00 or local equivalent; or lower extremity angiography: ICD9: 88.48,75710,75711,75712,75716,75717,75718,75630,75631: OPCS4 L63,L71,L72,U117 AND Z38,or local equivalent and a concurrent non-coronary vessel stent ICD9 39.50,39.90,37205,37206,37207,37208,37184,37185,37186; ICD10 L76,L98 or local equivalent; or a procedure code for lower extremity artery surgical and percutaneous vascular interventions: ICD9 : 38.18,39.50,39.25,39.29,38.08,38.38,38.48,39.49; 39.56,39.57,39.58,39.90,35302,35303,35304,35305,35306,35331,35351,35355,35361,35363,35371, 35372,35381,35452,35454,35456,35459,35470,35472,35473,35474,35481,35482,35483,35485,35491,35492,35493,35495,35521,35533,35537,35538,35539,35540,35541,35546,35548,35549,35551,35556,35558,35563,35565,35566,35571,35582,35583,35585,35587,35621,35623,35637,35638,35641, 35646,35647,35651,35654,35656,35661,35663,35665,35666,35671,35226,35256,35286,35700,35721,35741,35876,35879,35881,35883,35884,37184,37185,37186,37205,37206,37207,37208; OPCS4 L51-,L52,L531,L532,L538,L539,L54,L59-,L60-,L62-,L66-,L68-,L70-,Z38- or local equivalent excluding other reasons for surgery: ICD9: 736.3×,736.4×,736.5,736.6,736.7×,736.8×,736.9,735.×,754.3×,754.4×,754.5×,754.6×,754.7×,755.02,755.13,755.14,755.3,755.4,755.6×,755.8,759.7,759.89,895.××,896.××, 897.××,820.××,821.××,822.××,823.××,824.××,825.××,826.××,827.××,828.××,829.××,835.××,836.××,837.××,838.××,904.××,928.××,929.××,959.6,959.7,996.4×,996.66,996.67,996.77,996.78; ICD10:

M201,M202,M203,M204,M205,M206,M214,M216,M217,M219,M22-,M23-,M2406,M2416,M2426,M2436,M2446,M2456,M2466,M2476,M2476,M2486,M2496,M2506,M2516,M2526,M2536,M2546,M2556,M2566,M2576,M2586,M2596,M8406,M8416,M8426,M8436,M8446,M8456,M8466,M8476,M8486,M8496,Q682,Q683,Q684,Q685,Q65-,Q6-,Q692,Q702,Q703,Q704,Q72-,Q741,Q742,S72-,S730-,S75-,S77-,S78-,S797,S81-,S82-,S83-,S84-,S85-,S86-,S87-,S88-,S89-,S91-,S92-,S93-,S94-,S95-,S96-,S97-,S98-,T013,T016,T023,T025,T026,T033,T034,T043,T044,T047,T053,T054,T055,T056,T8416,T8426,T8436,T8446,T8456,T8466,T8476,T8486,T8496 or local equivalent; or amputations from thigh to mid foot: ICD9: 84.1×,84.91,27295,27590,27591,27592,27598,27880,27781,27782,27888,27889,28800,28805. ICD10: X09,X10,X11 or local equivalent excluding non-vascular amputations: ICD9: 736.3×,736.4×,736.5,736.6,736.7×,736.8×,736.9,735.×,754.3×,754.4×,754.5×,754.6×,754.7×,755.02,755.13,755.14,755.3,755.4,755.6×,755.8,759.7,759.89, 895.××, 896.××, 897.××,820.××,821.××,822.××,823.××,824.××,825.××,826.××,827.××,828.××,829.××,835.××,836.××,837.××,838.××,904.××,928.××,929.××,959.6,959.7,996.4×,996.66,996.67,996.77,996.78; ICD10: M201,M202,M203,M204,M205,M206,M214,M216,M217,M219,M22-,M23-,M2406,M2416,M2426,M2436,M2446,M2456,M2466,M2476,M2476,M2486,M2496,M2506,M2516,M2526,M2536,M2546,M2556,M2566,M2576,M2586,M2596,M8406,M8416,M8426,M8436,M8446,M8456,M8466,M8476,M8486,M8496,Q682,Q683,Q684,Q685,Q65-,Q6-,Q692,Q702,Q703,Q704,Q72-,Q741,Q742,S72-,S730-,S75-,S77-,S78-,S797,S81-,S82-,S83-,S84-,S85-,S86-,S87-,S88-,S89-,S91-,S92-,S93-,S94-,S95-,S96-,S97-,S98-,T013,T016,T023,T025,T026,T033,T034,T043,T044,T047,T053,T054,T055,T056,T8416,T8426,T8436,T8446,T8456,T8466,T8476,T8486,T8496 or local equivalent; or a prescription for medication used to treat claudication: Clisotazol, Pentoxifylline, Naftidrofuryl, Inositol nicotinate, Tymoxamine or their synonyms.

Controls: Individuals not meeting the case criteria and those individuals with any amputations below the mid-thigh. Individuals with any history of vascular disease were also excluded.

Although this is a longitudinal study, PAD cases and controls were analysed as a cross sectional study.

Heart Protection Study (HPS)

The Medical Research Council / British Heart Foundation Heart Protection Study (HPS) was a large randomized trial involving individuals at increased risk of vascular events. Between 1994-1997 20,536 men and women aged 40-80 years were recruited from 69 collaborating hospitals in the UK (with ethics committee approval). Participants were eligible for inclusion provided they had non-fasting blood total cholesterol concentrations of at least 135 mg/dL (3.5 mmol/L) and either a previous diagnosis of coronary disease, ischemic stroke, other occlusive disease of non-coronary arteries, diabetes mellitus, or (if were men 65 years or older) treated hypertension.

Cases: Cases of PAD were defined as those with a history of one or more of the following: peripheral or aortic revascularization, an amputation for vascular disease, or other evidence of PAD (such as IC) at entry into the study. Overall, there were 1,237 PAD cases.

Controls: Population controls were used from the UK Twins Study and Wellcome Trust Case Control Consortium 2 National Blood Service collections ($n_{controls}$=2,757).

Mayo Clinic

The Mayo Vascular Diseases Biorepository (VDB) was initiated inn 2006 and includes DNA and plasma samples of ~8000 patients with atherosclerotic cardiovascular disease (ASCVD) and 2203 age- and sex-matched controls without ASCVD. Participants were seen for their clinical care at Mayo Clinic, Rochester, MN, provided broad consent for genomic studies and samples are linked to the Mayo electronic health record (EHR). The ASCVD subtypes include PAD, abdominal aortic aneurysm, cerebrovascular disease, carotid artery stenosis and CAD.

Patient-level data elements in the EHR included demographics, outpatient visits and hospitalizations, providers, diagnosis and procedure codes, and results of non-invasive vascular evaluation. Birth date,

race, sex, and ethnicity were obtained from the demographic database. The majority (>95%) of the patients are non-Hispanic whites, reflective of the geographic location of the Mayo Clinic in Rochester, MN. We used validated electronic phenotyping algorithms developed as part of the eMERGE network, to ascertain cardiovascular risk factors. EHR-based algorithms had high specificity and sensitivity; the precision ranged from 0.93-1.00, and recall ranged from 0.75-0.99 for diabetes, hypertension, and smoking status. Genotypes were measured using high-density SNP arrays on two Illumina platforms[36–38].

Cases: The PAD patients in VDB were recruited from the non-invasive vascular laboratory at the Mayo Clinic Rochester, MN, based on the following criteria: 1) an ABI of 0.9 at rest or 1 min after exercise, along with an abnormal continuous wave Doppler signal in one of the lower extremity arteries; 2) history of lower extremity revascularization if the ABI was normal; and 3) ABI>1.4 or ankle systolic BP >250 mm Hg, representing poorly compressible arteries. Exclusion criteria included PAD secondary to vasculitis, radiation to the abdomen or lower extremities, trauma to lower extremity artery, thrombophilia, and arterial thrombosis.

Controls: Were identified from patients referred to the Cardiovascular Health Clinic for exercise ECG to screen for cardiovascular disease. We excluded patients who had a positive exercise ECG, were younger than age 50, or had an abnormal ABI or history of PAD. A proportion (60%) of the individuals who underwent exercise ECG also underwent measurement of ABI. The prevalence of an abnormal ABI in patients who had a negative stress ECG was <1%.

Scannia Diabetes Registry

Patients in Scannia Diabetes Registry (SDR) were randomly collected from the Department of Endocrinology, Malmö Sweden and surrounding clinics in Skåne (Scania) Sweden 3. The total cohort included 7414 individuals with all types of diabetes. Diabetes classification into T1D and T2D was done based on presence of GAD antibodies and c-peptide levels, or in case of incomplete information, based on the diagnosis given by the treating physician. Patients were selected for genotyping based on

presence of complications (kidney disease or retinopathy) or absence of complications in spite of more than 15/10 years duration of diabetes for T1D/T2D respectively[3]. Patients of known non-Scandinavian origin were excluded from the analysis.

Cases: PAD was diagnosed by an ankle brachial pressure index <0.9, or abnormal toe systolic pressures, pulse volume recordings, transcutaneous oxygen measurements or vascular imaging (e.g. with duplex ultrasound), or hospital admission or death for diabetes with peripheral circulatory disease (ICD10 codes; E11.5, or I73.9 or I74.3 or I 74.4 or ICD9 code 443), or prior corrective surgery, angioplasty, or above ankle amputation to the extremities.

Controls: Individuals who met the criteria of a PAD case or with any history of vascular disease were excluded from the controls.

The UK Biobank

UK Biobank (UKBB) recruited 500,000 people aged between 40-69 years in 2006-2010. They have undergone measures, provided blood, urine and saliva samples for future analysis, detailed information about themselves and agreed to have their health followed.

Cases: PAD was defined using questionnaire and electronic medical record data and restricted to individuals of European descent. The same ICD and OPCS codes that were used to define cases in GoDARTS were applied to the electronic medical record data available for the UKBB. Prescribing data were available from the questionnaire data so medication to treat claudication (Clisotazol, Pentoxifylline, Naftidrofuryl, Inositol nicotinate, Tymoxamine or their synonyms.) were also indicative of case status. UKBB participants also reported on other illnesses and operations in the questionnaire. PAD cases were defined as those reporting the following non-cancer illness codes: 1067 (peripheral vascular disease) and 1087 (leg claudication/IC) but excluded 1068 (venous thromboembolic disease), 1372 (vasculitis), 1546 (essential thrombocytosis) and 1088 (arterial embolism). Information was also available on surgical operations, from the questionnaire data, and the following codes were used to

define cases: 1440 (amputation of leg); 1441 (amputation of foot); 1102 (fem-pop bypass/leg artery bypass); and 1108 (leg artery angioplasty +/- stent).

Controls: The control definition was the same as GoDARTS and excluded individuals with any history of vascular disease.

Although there is some longitudinal data available through the EMR, the cohort was analysed as a cross sectional study.

The Wisconsin Epidemiologic Study of Diabetic Retinopathy: XVII

The population consisted of persons with type 1 diabetes who were receiving care in 11 counties in Wisconsin. Subjects (n = 996) were examined at baseline (1980-1982), and 4, 10, 14, and 20 years later. Evaluations included medical history and measurements of height, weight, blood pressure, and glycosylated hemoglobin. Fundus photographs were graded for diabetic retinopathy at baseline, and the same photographs were graded later for the diameters of retinal blood vessels. At each examination, a history of cardiovascular disease events since the last examination (and prior to baseline) was obtained. Mortality was monitored yearly.

Cases: PAD was defined as a clinical record of an ABI at rest or post exercise of <0.9 and/or an ankle systolic blood pressure >255mmHg (excluding any non-atherosclerotic causes of PAD) and/or amputations from the thigh through to midfoot (excluding any non-vascular amputations).

Controls: No leg amputations regardless of whether above or below ankle; and no history of PAD surgery; and no known clinical diagnosis of PAD; and no known prescriptions for medication used to treat claudication

Genome-wide association studies

Eleven studies identified PAD cases and controls and performed logistic regression analyses corrected for age, gender and study specific covariates whilst taking genotype uncertainty into account. The specific analysis details and software used by individual studies are given in Supplemental Table II. When diabetes status or smoking status was available additional logistic regression analyses of PAD

corrected for age and gender were performed in 1) Individuals classified as ever smokers; 2) Individuals classified as never smokers; 3) Individuals with no history of diabetes and; 4) Individuals with diabetes. Summary statistics from individual studies were restricted to variants with a minor allele frequency > 1%, Hardy-Weinberg equilibrium (HWE) test $p>1\times10^{-6}$, call rate > 98% for directly typed variants and imputation information score > 0.4 for imputed variants.

Meta-analysis

Individual study summary statistics were combined separately for each of the five PAD GWAS-meta-analyses (i.e., primary, ever smokers, never smokers, without diabetes and with diabetes) in a fixed-effects inverse variance-weighting scheme using GWAMA (Genome-Wide Association Meta-Analysis) software v2.2.2[15]. The primary meta-analysis included all available samples without stratification by smoking or diabetes status whilst the stratified analyses were based on analysing ever smokers, never smokers, individuals without diabetes and with diabetes separately and combining in a smoking or diabetes genome-wide interaction analysis respectively.

We also combined allelic effects for the 19 published variants from the MVP[11] with allelic effects estimated in GoLEAD excluding the UKBB from the GoLEAD meta-analysis. This was done as the MVP meta-analysis included the UKBB as their replication sample. Between study heterogeneity was calculated using Cochran's Q test and I^2.[39] After the summary statistics were meta-analysed the variants were restricted to those supported by >2 studies and an effective sample size (N_{eff} $= \frac{4}{(\frac{1}{N_{cases}}+\frac{1}{N_{controls}})} = \frac{4}{(\frac{1}{N_{cases}}+\frac{1}{N_{controls}})}$) >4000. Variants were tested for interaction with smoking and diabetes status by testing for heterogeneous allelic effects between smoking and diabetes strata separately[15]. Sensitivity analyses using a random effects model were also conducted.

Estimation of heritability

Narrow sense, chip heritability was estimated using LDScore regression from the primary PAD GWAS using a disease prevalence of 5% and estimated the heritability using the recommended settings[40]. The heritability estimates were comparable to GCTA estimates of chip heritability as the $N_{cases}>5000$[41].

LD regression scores were pre-computed in the 1000G reference panel (Phase 1) for individuals of European descent for all SNPs that were genotyped these are a function of R^2 and give a measure of the amount of genetic variation tagged by that SNP.

Conditional analysis

For all lead variants passing the genome-wide significance threshold of $p \le 5\times10^{-8}$, conditional analyses for other variants within a 1Mb region were performed using GCTA v1.91.1 (-cojo, conditional and joint analysis)[41]. The reference panel was generated from 410,715 unrelated (1st and 2nd degree relatives removed) UKBB participants of European descent. Variants in the reference panel were restricted to those within 500kb upstream and downstream of lead variants (those with the lowest p value in a 1Mb region) that had an imputation info score >0.80, and HWE P greater than 1×10^{-6} and a minor allele count ≥3. First, the region was conditioned on the lead variant and if additional signals that had an unconditioned $p \le 5\times10^{-8}$ also had a conditioned $p \le 5\times10^{-5}$ then this was counted as a second independent signal. The region was then conditioned on both variants to identify any additional independent associations in the region.

PhenoScanner

PhenoScanner, a database of publicly available results from GWAS[42], was used to search for other traits associated with our lead SNPs, or proxy SNPs in high LD ($r^2 > 0.8$) with a p-value of less than 5×10^{-5}. We limited our results to vascular disease or related traits; each study was selected based on the minimum p-value for the lead SNP (or proxy). All PhenoScanner results are shown for the PAD risk increasing allele.

Post hoc power calculations for individual SNP associations

To identify whether variants associated with PAD in the stratified analyses were stratum-specific or whether they represented differences in power to detect associations based on the different sample sizes we performed power calculations using CaTS[43]. For each variant detected at genome-wide significance with PAD in individuals without diabetes, we calculated the power to detect an association

at an $\alpha=5\times10^{-8}$ for that variant in individuals with diabetes. The power to detect the effect size, estimated in individuals without diabetes, was calculated using the sample size and allele frequency from individuals with diabetes. In a similar manner, we did the same for variants associated with PAD in individuals with diabetes at genome-wide significance, and for the two strata of PAD by smoking status.

Interaction analysis post hoc power calculation

Statistical interaction was calculated by testing the difference between two estimates of allelic effect on PAD. The power to detect an interaction depends on how accurately the allelic effect can be estimated in each stratum. We assessed the power to detect an interaction effect of a PAD-risk variant with diabetes and separately for smoking status under three allelic effect scenarios: a) an effect on PAD in subjects with diabetes/ever smokers only (i.e. OR is 1 in subjects without diabetes/never smokers, but varies between 1 and 1.2 in subjects with diabetes/ever smokers); b) an effect on PAD in subjects with diabetes/ever smokers and without diabetes/never smokers in the same direction but of differing magnitude (i.e. OR is 1.10 in subjects without diabetes/never smokers, but varies between 1 and 1.2 in subjects with diabetes/ever smokers); and c) an effect on PAD in subjects with diabetes/ever smokers and without diabetes/never smokers but in opposite directions (i.e. OR is 0.90 in subjects without diabetes/non-smokers, but varies between 1 and 1.2 in subjects with diabetes/ever smokers). For each scenario, we evaluated two risk allele frequencies: 10% and 50%. Power was calculated for $\alpha\leq5\times10^{-4}$ and separately for $\alpha\leq5\times10^{-7}$ based on the maximum available sample size for the diabetes or smoking stratified analyses separately.

Sample size calculation for replication of interactive effects

We performed a sample size estimation for replication of the interactive effects based on published population prevalence for diabetes, smoking and PAD in respective risk groups. The prevalence of diabetes in 2017 in the US was 9.8%[44] and the prevalence of PAD in subjects with diabetes is 20%[45], so in 1000 patients you would predict 98 of them would have diabetes (either T1D or T2D) and of those

98 approximately 20 of those would have PAD. Of the 902 remaining individuals 5% (45) of those are expected to have PAD leaving 857 without PAD and diabetes. The prevalence of PAD in heavy smokers is 9.8%[46], and we found in the UKBB that the number of ever vs never smokers was ~50% so we calculated the proportions based on these numbers. Based on this we determined the following proportions: individuals with diabetes and PAD=2%; with diabetes and no history of PAD=8%; individuals without diabetes and PAD=5%; individuals without diabetes and no history of PAD=85%; individuals that have ever smoked and had PAD=6%; individuals who ever smoked and with no history of PAD=45%; individuals who never smoked and had PAD=2%; and individuals who never smoked and had no history of PAD=47%. A replication was considered allelic effects in the same direction as the discovery population and a $p_{interaction}<0.05$ and $p_{interaction}<2\times10^{-4}$ (0.05/218[Number of loci reaching $p_{association}$ or $p_{interaction}<5\times10^{-5}$ in the diabetes interaction analysis]).

Genetic correlation analyses

The genetic correlations of PAD and CAD with known risk factors: body mass index (BMI)[20]; low-density lipoprotein cholesterol (LDL-C); high-density lipoprotein cholesterol (HDL-C); triglycerides (TGL)[21]; type 2 diabetes (T2D)[22]; and systolic blood pressure (SBP) (UKBB automated reading) were calculated based on overlapping variants between the GWAS datasets for the outcomes and the known risk factors with 1000G Europeans, using LDSC (v1.0.1)[4748].

Supplemental Tables

Supplemental Table I: Each study included in the meta-analysis defined cases of peripheral artery disease cases and controls free of PAD in different ways dependent on the data available in each study. The individual case and control definitions are given below.

Study	Full name	Case definition	Control definition	N cases/controls	P*	D	ND	ES	NS
Corogene	Corogene	A clinical record of an ankle brachial pressure index (ABPI) at rest or post exercise of <0.9 or surgical interventions related to PAD	Individuals with no history of PAD	243/1,901					
DCCT/EDIC	The Diabetes Control and Complications Trial / The Epidemiology of Diabetes Interventions	A clinical record of an ankle brachial pressure index (ABPI) at rest or post exercise of <0.9 or an ankle systolic blood pressure >255mmHg or amputations from thigh through to midfoot	No leg amputations regardless of whether above or below ankle; and no history of peripheral vascular disease surgery; and no known clinical diagnosis of PAD; and no known prescriptions for medication used to treat claudication	718/541					

Study	Full name	Case definition	Control definition	N cases/controls	P*	D	ND	ES	NS
	and Complications								
deCODE		A clinical diagnosis of PAD at the major hospital in Reykjavik, the Landsite University Hospital, during the years 1983 to 2006 from registry data. Diagnosis was confirmed by vascular imaging or segmental pressure measurements.	Individuals with no history of PAD	1,468/21,166					
EAS	Edinburgh artery study	ABPI<0.9; ankle systolic pressure > 255mmHg; amputations; surgical interventions related to PAD	Individuals with no history of PAD	130/634					
FinnDiane	The Finnish Diabetic Nephropathy Study	Major amputations (non-vascular causes excluded), surgical interventions related to PAD (peripheral artery bypass surgery, percutaneous angioplasty for peripheral arterial disease)	Controls without any known PAD (no amputations, no surgical interventions related to PAD), type 1 diabetes duration >10 yrs.	303/2,520					

Study	Full name	Case definition	Control definition	N cases/controls	P*	D	ND	ES	NS
GoDARTS	The Genetics of Diabetes Audit and Research in Tayside Scotland	PAD case was defined as a clinical record of an ABPI at rest or post exercise of < 0.9 or an ankle systolic blood pressure of > 255 mmHg or an abnormal toe pressure index of < 0.7 or an abnormal toe pressure < 65 mmHg. The definition also included hospital admission codes for PAD or a procedure code for a lower extremity angiography and a concurrent code for non-coronary vessel stent, or a procedure code for lower extremity artery surgery excluding alternate reasons for surgery, or a procedure code for lower extremity percutaneous vascular intervention excluding alternate reasons, or mid-thigh to mid-foot amputations excluding non-vascular amputations or a prescription for medication used to treat claudication. PAD controls excluded all individuals who met the case criteria and excluded all those with amputation, or any PAD related surgeries. Dispensed medication to treat claudication was also included indicative of case status. Controls excluded all individuals meeting the case definition plus any amputation in the lower limbs.	Controls free of any vascular disease	1,223/5,639					
HPS	MRC/BHF Heart Protection Study	Yes, to having either: Other arterial bypass surgery, Amputation for vascular disease or other evidence of peripheral vascular disease. Measured at baseline	Population controls from WTCCC2 National Blood Service Collections and UK Twins Study (population prevalence of PAD)	1,237 / 2,757					

Study	Full name	Case definition	Control definition	N cases/controls	P*	D	ND	ES	NS
		Category: Self-reported							

Study	Full name	Case definition	Control definition	N cases/controls	P*	D	ND	ES	NS
Mayo Clinic	Genetic Determinants of Peripheral Artery Disease	ABPI < 0.9 at rest or 1 min after exercise, along with an abnormal continuous wave Doppler signal in one of the lower extremity arteries; 2) history of lower extremity revascularization if the ABPI was normal; and 3) ABPI > 1.4 or ankle systolic BP >250 mm Hg	Controls were identified from patients referred to the Cardiovascular Health Clinic for exercise ECG to screen for cardiovascular disease. We excluded patients who had a positive exercise ECG, were younger than age 50, or had an abnormal ABI or history of PAD. A proportion (60%) of the subjects who underwent exercise ECG also underwent measurement of ABI. The prevalence of an abnormal ABI in patients who had a negative stress ECG was <1%.	2,707/2,046					

Study	Full name	Case definition	Control definition	N cases/controls	P*	D	ND	ES	NS
		Category: Clinical diagnosis							
SDR	Scannia Diabetes registry	i) A diagnosis of PAD that has been confirmed by an ABPI <0.9, OR abnormal toe systolic pressures, pulse volume recordings, transcutaneous oxygen measurements or vascular imaging (e.g. with duplex ultrasound)or<br>ii) hospital admission or death for diabetes with peripheral circulatory disease (ICD10 codes; E11.5, or I73.9 or I74.3 or I 74.4 or ICD 9 code 443) or<br>iii) prior corrective surgery, angioplasty, or ABOVE ANKLE amputation to the extremities.<br>Category: Clinical diagnosis	Individuals free of any vascular disease	274/2,065					

Study	Full name	Case definition	Control definition	N cases/controls	P*	D	ND	ES	NS
UKBB	UK Biobank	The definition also included hospital admission codes for PAD or a procedure code for a lower extremity angiography and a concurrent code for non-coronary vessel stent, or a procedure code for lower extremity artery surgery excluding alternate reasons for surgery, or a procedure code for lower extremity percutaneous vascular intervention excluding alternate reasons, or mid-thigh to mid-foot amputations excluding non-vascular amputations or a prescription for medication used to treat claudication. Peripheral artery disease controls excluded all individuals who met the case criteria and excluded all those with amputation, or any PAD related surgeries. Dispensed medication to treat claudication was also included indicative of case status. Controls excluded all individuals meeting the case definition plus any amputation in the lower limbs.<br>Category: Most cases were self-reported	Individuals free of any known vascular disease	3,677/409,939					
WESDR	Wisconsin Epidemiologic Study of Diabetic Retinopathy	A clinical record of an ABPI at rest or post exercise of <0.9 or • An ankle systolic blood pressure >255mmHg or • Amputations from thigh through to midfoot<br>Category Clinical diagnosis	No leg amputations regardless of whether above or below ankle; and no history of PAD surgery; and no known clinical diagnosis of PAD; and no known prescriptions for medication used to treat claudication	106/340					

*P primary analysis; D diabetes only; ND no history of diabetes; ES ever smoker; and NS never smoker. Green indicates that the study was included in the specific analysis while black indicates that it was excluded.

Supplemental Table II: Sample and genotype characteristics for the eleven studies included in the meta-analysis
This table is available in the Supplemental tables excel sheet and was too large to place here

Supplemental Table III: Independent variants associated ($p \leq 5\times10^{-8}$) with peripheral artery disease in the primary analysis were also associated with other traits

**TRAIT**		**PMID**	**SNP**	**LOCUS**	**PROXY SNP**	**PAD RISK ALLELE**	**PAD INCREASING ALLELE FOR PROXY SNP**	**TRAIT RISK / LEVELS**
**PERIPHERAL ARTERIAL DISEASE (PAD)**	PAD	18385739	rs1317286	*CHRNA5*	rs1051730	G	A	Increase
	PAD	25009551	rs10774624	*SH2B3*	rs653178	G	C	Increase
	Ankle brachial index	22199011	rs10738610	*CDKN2A*	rs1537375	C	C	Decrease
**CORONARY HEART DISEASE (CHD)**	CHD / MI	26343387	rs10738610	*CDKN2A*	rs2891168	C	G	Increase
	CHD / MI	26343387	rs10455872	*LPA*	rs55730499	G	T	Increase
	CHD / MI	26343387	rs10774624	*SH2B3*	rs653178	G	C	Increase
	CHD / MI	23202125	rs11066309	*PTPN11*	rs17630235	A	A	Increase
	CHD / MI	23202125	rs7452960	*LPA*	rs3798220	A	C	Increase
**ISCHAEMIC STROKE**	Ischaemic stroke	23041239	rs11066309	*PTPN11*	rs17696736	A	G	Increase
	Large artery stroke	24262325	rs10738610	*CDKN2A*	rs1333047	C	T	Increase
**ABDOMINAL AORTIC ANEURYSM (AAA)**	AAA	20622881	rs10738610	*CDKN2A*	rs2383207	C	G	Increase
**DIABETES**	Diabetes Type 1	21829393	rs10774624	*SH2B3*	rs3184504	G	T	Increase

TRAIT		PMID	SNP	LOCUS	PROXY SNP	PAD RISK ALLELE	PAD INCREASING ALLELE FOR PROXY SNP	TRAIT RISK / LEVELS
KIDNEY DISEASE	Diabetes Type 1	18978792	rs11066309	*PTPN11*	rs17696736	A	G	Increase
	Diabetes Type 2	17554300	rs10738610	*CDKN2A*	rs1333049	C	C	Increase
	Chronic kidney disease	20383146	rs10774624	*SH2B3*	rs653178	G	C	Increase
	Urate levels	23263486	rs10774624	*SH2B3*	rs653178	G	C	Increase
	eGFR cystatin C	26831199	rs10774624	*SH2B3*	rs653178	G	C	Decrease
	eGFR cystatin C	26831199	rs11066309	*PTPN11*	rs11066188	A	A	Decrease
	eGFR creatinine (in non-diabetics)	26831199	rs10455872	*LPA*		G		Decrease
LIPIDS	Lp(a)	20032323	rs10455872	*LPA*		G		Increase
	Lp(a)	20032323	rs7452960	*LPA*	rs3798220	A	C	Increase
	Total cholesterol	20686565	rs10455872	*LPA*		G		Increase
	Total cholesterol	24097068	rs10774624	*SH2B3*	rs3184504	G	T	Decrease
	Total cholesterol	24097068	rs11066309	*PTPN11*	rs17630235	A	A	Decrease
	Total cholesterol	24097068	rs7452960	*LPA*	rs3798220	A	C	Increase

TRAIT		PMID	SNP	LOCUS	PROXY SNP	PAD RISK ALLELE	PAD INCREASING ALLELE FOR PROXY SNP	TRAIT RISK / LEVELS
	LDL cholesterol	23063622	rs10455872	*LPA*		G		Increase
	LDL cholesterol	24097068	rs10774624	*SH2B3*	rs3184504	G	T	Decrease
	LDL cholesterol	24097068	rs11066309	*PTPN11*	rs17630235	A	A	Decrease
	LDL cholesterol	24097068	rs7452960	*LPA*	rs3798220	A	C	Increase
	LDL cholesterol response to statins	23100282	rs10455872	*LPA*		G		Decrease
	HDL cholesterol	24097068	rs10774624	*SH2B3*	rs653178	G	C	Decrease
	HDL cholesterol	24097068	rs11066309	*PTPN11*	rs17630235	A	A	Decrease
	Apolipoprotein B response to statins	23100282	rs10455872	*LPA*		G		Decrease
	Apolipoprotein B response to statins	23100282	rs7452960	*LPA*		A		Decrease
**HYPERTENSION**	Diastolic blood pressure	21909115	rs10774624	*SH2B3*	rs3184504	G	T	Increase
	Diastolic blood pressure	21909115	rs11066309	*PTPN11*	rs17630235	A	A	Increase

TRAIT		PMID	SNP	LOCUS	PROXY SNP	PAD RISK ALLELE	PAD INCREASING ALLELE FOR PROXY SNP	TRAIT RISK / LEVELS
	Systolic blood pressure	21909115	rs11066309	*PTPN11*	rs17630235	A	A	Increase
	Systolic blood pressure	21909115	rs10774624	*SH2B3*	rs3184504	G	T	Increase
	Mean arterial pressure	21909110	rs10774624	*SH2B3*	rs3184504	G	T	Increase
**ADIPOSITY**	BMI / hip circumferenc e	26426971	rs11066309	*PTPN11*	rs17630235	A	A	Decrease
	BMI / hip circumferenc e	25673413	rs10774624	*SH2B3*	rs3184504	G	T	Decrease
	BMI	26426971	rs1317286	*CHRNA5*	rs7180002	G	T	Decrease
**SMOKING**	Daily smoking	22832964	rs1317286	*CHRNA5*	rs1051730	G	A	Increase
	COPD	24621683	rs1317286	*CHRNA5*	rs12914385	G	T	Increase

Supplemental Table IV: Combined association results from Genetics of Lower Extremity Arterial disease (GoLEAD), Million Veterans Program (MVP) and United Kingdom Biobank (UKBB) for 19 published variants associated with peripheral artery disease.

Chrom:pos*	SNP (nearest Gene)	EA†/ NEA‡ (EAF§)	GoLEAD (inclu. UKBB)					GoLEAD (exclu. UKBB) and MVP&UKBB				
			OR (95%CI)	*p*	Q*VALUE*	I^2	Cases/ controls	OR (95%CI)	*p*	Q*VALUE*	I^2	Cases/ controls
1:109817192	rs7528419 (*CELSR2/SORT1*)	A/G (0.78)	1.05 (1.01,1.08)	0.01	0.13	0.56	12085/ 449540	1.06 (1.05,1.08)	$2.1{\times}10^{-12}$	0.68	0.00	44832/ 640645
1:169519049	rs6025 (*F5*)	T/C (0.97)	1.14 (1.04,1.25)	0.02	1.00		8613/ 417348	1.20 (1.15,1.25)	$7.1{\times}10^{-13}$	0.94	0.00	41360/ 608453
6:160985526	rs118039278 (*LPA*)	A/G (0.07)	1.22 (1.13,1.31)	$6.2{\times}10^{-10}$	0.24	0.27	10245/ 425851	1.26 (1.21,1.31)	$6.5{\times}10^{-50}$	0.13	0.35	42992/ 616956
6:31065071	rs3130968 (*HLA-B*)	T/C (0.19)	1.05 (0.99,1.11)	0.12	1.00		4395/ 410480	1.07 (1.05,1.09)	$2.7{\times}10^{-12}$	0.52	0.00	37142/ 601585
7:19049388	rs2107595 (*HDAC9*)	A/G (0.83)	1.11 (1.07,1.15)	$1.5{\times}10^{-7}$	0.90	0.00	11956/ 448913	1.09 (1.06,1.11)	$4.2{\times}10^{-11}$	0.47	0.00	44703/ 640018
7:22786532	rs4722172 (*IL6)*	G/A (0.78)	1.05 (1.01,1.09)	0.01	0.57	0.00	10245/ 425851	1.08 (1.05,1.10)	$8.8{\times}10^{-9}$	0.66	0.00	42992/ 616956
8:19819217	rs322 (*LPL*)	A/C (0.27)	1.06 (1.02,1.09)	$3.0{\times}10^{-3}$	0.37	0.00	10245/ 425851	1.06 (1.04,1.07)	$1.5{\times}10^{-9}$	0.76	0.00	42992/ 616956
9:136149229	rs505922 (*ABO*)	C/T (0.66)	1.08 (1.05,1.11)	$2.0{\times}10^{-6}$	0.88	0.00	11020/ 449096	1.06 (1.04,1.08)	$1.2{\times}10^{-10}$	0.67	0.00	43767/ 640201
9:22103183	rs1537372 (*CDKN2B-AS1*)	T/G (0.42)	1.11 (1.07,1.15)	$1.1{\times}10^{-10}$	0.40	0.00	10375/ 426485	1.12 (1.10,1.14)	$6.8{\times}10^{-41}$	0.18	0.28	43122/ 617590
10:114758349	rs7903146 (*TCF7L2*)	T/C (0.71)	1.03 (1.00,1.06)	0.08	0.29	0.09	11020/ 449108	1.06 (1.04,1.07)	$4.9{\times}10^{-10}$	0.07	0.40	43767/ 640213
11:102710471	rs566125 (*MMP3*)	T/C (0.86)	1.04 (1.00,1.09)	0.06	1.00	0.00	11955/ 448911	1.07 (1.04,1.09)	$2.6{\times}10^{-7}$	0.76	0.00	44702/ 640016

Chrom:pos*	SNP (nearest Gene)	EA†/ NEA‡ (EAF§)	GoLEAD (inclu. UKBB) OR (95%CI)	*p*	Q_{VALUE}	I^2	Cases/ controls	GoLEAD (exclu. UKBB) and MVP&UKBB OR (95%CI)	*p*	Q_{VALUE}	I^2	Cases/ controls
11:46342834	rs7476 (*CREB3L1*)	C/A (0.69)	1.01 (0.98,1.04)	0.55	0.53	0.00	11955/ 448900	1.05 (1.03,1.07)	1.2×10^{-8}	0.60	0.00	44702/ 640005
12:112871372	rs11066301 (*SH2B2-PTPN11*)	G/A (0.57)	1.08 (1.05,1.11)	8.7×10^{-7}	0.96	0.00	11020/ 449109	1.06 (1.05,1.08)	3.1×10^{-12}	0.01	0.56	43767/ 640214
12:79951566	rs4842266 (RP11-359M6.3)	G/A (0.32)	1.04 (1.01,1.08)	0.02	0.17	0.47	11243/ 446099	1.06 (1.04,1.08)	1.4×10^{-9}	0.47	0.00	43990/ 637204
13:110828891	rs1975514 (*COL4A1*)	C/T (0.36)	1.04 (1.00,1.07)	0.02	0.45	0.00	11243/ 446099	1.05 (1.04,1.06)	1.6×10^{-23}	0.48	0.00	43990/ 637204
14:70501364	rs55784307 (*SMOC1*)	A/C (0.19)	1.07 (1.03,1.12)	1.1×10^{-3}	0.68	0.00	10245/ 425851	1.06 (1.04,1.08)	1.2×10^{-10}	0.53	0.00	42992/ 616956
15:78915864	rs10851907 (*CHRNA3*)	A/G (0.41)	1.08 (1.04,1.11)	1.2×10^{-5}	0.65	0.00	10245/ 425851	1.06 (1.05,1.07)	1.2×10^{-35}	0.61	0.00	42992/ 616956
17:66089393	rs62084752 (LOC732538)	C/G (0.75)	1.08 (1.04,1.12)	2.2×10^{-4}	0.77	0.00	9008/ 423094	1.07 (1.05,1.09)	9.1×10^{-13}	0.07	0.44	41755/ 614199
19:11191729	rs138294113 (*LDLR*)	C/T (0.11)	1.09 (1.04,1.14)	5.7×10^{-4}	0.27	0.17	10245/ 425851	1.09 (1.06,1.11)	7.2×10^{-10}	0.90	0.00	42992/ 616956

*Chrompos: Chromosome number and position; †EA: Effect allele; ‡NEA: Non-effect allele; §EAF: Effect allele frequency

Supplemental Table V: There are eleven previously reported variants associated with peripheral artery disease (PAD). This table shows the association of these variants in our primary, diabetes and smoking stratified analyses where the allelic effects were aligned to the published PAD risk allele.

CHR	BP	SNP (Gene) PMID	Reported			GoLEAD study					
			EA	EAF	OR	EAF	Phenotype	OR (95%CI)	P	#cases	#controls
4	148620269	rs6842241 (*EDNRA*) East Asian 26488411	C	0.70	1.18	0.86	Primary	0.94 (0.90,0.98)	$3.4×10^{-3}$	12,080	449,434
						0.86	In individuals with diabetes	0.95 (0.88,1.01)	0.14	3,845	28,879
						0.86	In individuals without diabetes	0.94 (0.90,0.99)	0.03	6,722	416,740
						0.86	In ever smokers	0.93 (0.89,0.98)	$7.8×10^{-3}$	7,400	205,617
						0.86	In never smokers	0.97 (0.90,1.05)	0.53	2,414	239,769
6	160930108	rs10455872 (*LPA*) 21252144	G	0.09	1.14	0.06	Primary	1.23 (1.19,1.28)	$2.4×10^{-12}$	10,876	447,454
						0.06	In individuals with diabetes	1.25 (1.09,1.43)	$1.2×10^{-4}$	3,735	28,478
						0.08	In individuals without diabetes	1.23 (1.12,1.34)	$1.2×10^{-7}$	6,650	416,228
						0.07	In ever smokers	1.30 (1.22,1.37)	$7.1×10^{-11}$	6,978	203,771
						0.07	In never smokers	1.12 (1.01,1.24)	0.06	2,183	231,555
7	19002445	rs2074633 (*HDAC9*) East Asian	G	0.38	1.16	0.22	Primary	1.09 (1.05,1.12)	$6.0×10^{-6}$	11,956	448,909
						0.23	In individuals with diabetes	0.99 (0.93,1.05)	0.72	3,831	28,830
						0.21	In individuals without diabetes	1.13 (1.09,1.18)	$3.5×10^{-7}$	6,613	416,263

CHR	BP	SNP (Gene) PMID	Reported			GoLEAD study					
			EA	EAF	OR	EAF	Phenotype	OR (95%CI)	P	#cases	#controls
		26488411				0.22	In ever smokers	1.08 (1.04,1.13)	6.4×10^{-4}	7,310	205,330
						0.23	In never smokers	1.07 (1.01,1.15)	0.06	2,378	239,545
9	22062264	rs10757269 (*CDKN2BAS1*) 22199011	G	0.49	1.08	0.46	Primary	1.11 (1.07,1.14)	1.2×10^{-11}	11,956	448,916
						0.46	In individuals with Diabetes	1.12 (1.06,1.19)	2.2×10^{-5}	3,831	28,830
						0.47	In individuals without Diabetes	1.08 (1.03,1.12)	1.6×10^{-4}	6,650	416,234
						0.47	In ever smokers	1.13 (1.08,1.18)	3.8×10^{-10}	7,310	205,334
						0.46	In never smokers	1.02 (0.96,1.09)	0.44	2,378	239,549
12	110368991	rs3184504 (*SH2B3*) 25009551	T	0.46	1.22	0.48	Primary	1.09 (1.06,1.12)	2.1×10^{-8}	11,009	449,059
						0.49	In individuals with diabetes	1.06 (1.01,1.12)	0.03	3,646	28,704
						0.46	In individuals without diabetes	1.09 (1.06,1.13)	6.8×10^{-6}	6,084	416,424
						0.47	In ever smokers	1.08 (1.04,1.11)	1.7×10^{-4}	6,527	205,399
						0.48	In never smokers	1.11 (1.06,1.17)	6.4×10^{-4}	2,217	239,612
12	110492139	rs653178 (*SH2B3*) 25009551	C	0.47	1.22	0.48	Primary	1.09 (1.06,1.12)	2.6×10^{-8}	10,307	446,289
						0.49	In individuals with diabetes	1.07 (1.01,1.13)	0.03	2,936	25,886
						0.46	In individuals without diabetes	1.09 (1.05,1.13)	1.5×10^{-5}	6,092	416,472

CHR	BP	SNP (Gene) PMID	Reported			GoLEAD study					
			EA	EAF	OR	EAF	Phenotype	OR (95%CI)	P	#cases	#controls
						0.47	In ever smokers	1.08 (1.04,1.11)	1.7×10^{-4}	6,535	205,428
						0.48	In never smokers	1.11 (1.05,1.17)	1.1×10^{-3}	2,217	239,627
13	97161483	rs9584669 (*IPO5*) East Asian 26488411	T	0.94	1.72	0.87	Primary	1.02 (0.97,1.06)	0.48	11,928	448,905
						0.89	In individuals with diabetes	1.02 (0.94,1.10)	0.71	3,831	28,819
						0.85	In individuals without diabetes	1.01 (0.95,1.06)	0.80	6,601	416,254
						0.86	In ever smokers	1.03 (0.98,1.09)	0.26	7,284	205,329
						0.89	In never smokers	1.02 (0.93,1.11)	0.71	2,376	239,542
15	76681394	rs1051730 (*CHRNA3*) 18385739	T	0.35	1.19	0.33	Primary	1.09 (1.06,1.12)	4.9×10^{-8}	12,086	449,533
						0.33	In individuals with diabetes	1.03 (0.98,1.09)	0.27	3,846	28,880
						0.34	In individuals without diabetes	1.12 (1.09,1.16)	8.7×10^{-9}	6,728	416,837
						0.33	In ever smokers	1.12 (1.08,1.16)	1.1×10^{-8}	7,404	205,686
						0.33	In never smokers	1.03 (0.97,1.09)	0.44	2,414	239,799

Supplemental Table VI: Two additional known loci were associated with peripheral arterial disease in individuals without diabetes at p≤5×10^{-8}

CHR	BP	SNP (Gene)	EA	NEA	EAF	OR(95%CI)	P	P_{het}	#cases	#controls
12	111833788	rs10774624 (*CDKN2BAS*1)	G	A	0.49	1.14 (1.10,1.19)	2.3×10^{-9}	0.06	5,315	395,290
15	78826180	rs931794 (*CHRNA5*)	G	A	0.34	1.14 (1.09,1.19)	3.7×10^{-10}	1.4×10^{-3}	6,650	416,235

Supplemental table VII: Four known loci represented by four lead variants near *LPA, CDKN2BAS1* and *CHRNA5* were associated with peripheral arterial disease in ever smokers including a novel locus,*HLA-DRB1*,led by rs200841208.

CHR	BP	SNP (Gene)	EA	NEA	EAF	OR(95%CI)	P	P_{het}	#cases	#controls
6	161010118	rs10455872 (*LPA*)	G	A	0.07	1.30 (1.22,1.37)	7.1×10^{-11}	0.05	6,978	203,771
6	32537884	rs200841208 (*HLA-DRB2*)	T	TGC	0.30	1.35 (1.18,1.55)	3.6×10^{-8}	2.3×10^{-4}	2,392	5,054
9	22088260	rs10757272 (*CDKN2BAS1*)	T	C	0.46	1.15 (1.11,1.20)	1.1×10^{-13}	4.8×10^{-3}	7,310	205,334
15	78891627	rs12910984 (*CHRNA3*)	A	G	0.76	1.15 (1.11,1.19)	9.3×10^{-10}	3.9×10^{-5}	7,404	205,696

Supplemental Table VIII: We identified eight variants associated with PAD in diabetes and smoking stratified analyses. We calculated the power to detect these variants in the corresponding stratum given the allelic effects of the discovery, a disease prevalence of 5%, $\alpha=5\times10^{-8}$, the effect allele frequency and sample size in the corresponding stratum.

CHR	BP	SNP (Gene)	EA	EAF	OR(95%CI)	P	#cases/ #controls	Stratum	Power	#cases/ #controls	Power	Stratum
7	19049388	rs2107595 (*HDAC9*)	A/G	0.16	1.16 (1.11,1.21)	3.6×10^{-8}	6,616/ 416,267	Without diabetes	0.21	3,831/ 28,829	0.13	With diabetes
12	111833788	rs10774624 (*CDKN2BAS1*)	G/A	0.49	1.14 (1.10,1.19)	2.3×10^{-9}	5,315/ 395,290	Without diabetes	0.46	3,846/ 28,881	0.13	With diabetes
15	78826180	rs931794 (*CHRNA5*)	G/A	0.34	1.14 (1.09,1.19)	3.7×10^{-10}	6,650/ 416,235	Without diabetes	0.39	3,846/ 28,881	0.13	With diabetes
6	161010118	rs10455872 (*LPA*)	G/A	0.07	1.30 (1.22,1.37)	7.1×10^{-11}	6,978/ 203,771	Ever smokers	0.25	2,414/ 239,806	0.01	Never smokers
6	32537884	rs200841208 (*HLA-DRB1*)	T/TGC	0.30	1.35 (1.18,1.55)	3.6×10^{-8}	2,392/ 5,054	Ever smokers	1.00	751/ 3,975	0.01	Never smokers
9	22088260	rs10757272 (*CDKN2BAS1*)	T/C	0.46	1.15 (1.11,1.20)	1.1×10^{-13}	7,310/ 205,334	Ever smokers	0.24	2,414/ 239,806	0.01	Never smokers
15	78891627	rs12910984 (*CHRNA3*)	A/G	0.76	1.15 (1.11,1.19)	9.3×10^{-10}	7,404/ 205,696	Ever smokers	0.06	2,414/ 239,806	0.01	Never smokers
4	91588354	rs116405693 (*CCSER1*)	T/C	0.04	1.51 (1.32,1.74)	2.5×10^{-9}	3,454/ 26,707	With diabetes	1.00	5,315/ 395,290	0.02	Without diabetes

***Supplemental table IX*: ** The number of cases and controls required in each stratum to achieve >80% power to detect an interaction where the OR=1.5 in one stratum and no effect in the other stratum.

**Stratum**	***p*=0.05**			***p*=2×10^{-4}**		
	**N_{cases}**	**$N_{controls}$**	**Total**	**N_{cases}**	**$N_{controls}$**	**Total**
Diabetes	840	3,360	42,000	2,320	9,280	116,000
No history of diabetes	2,100	35,700		5,800	98,600	
Ever smokers	2,400	18,000	40,000	6,300	47,250	105,000
Never smokers	800	18,800		2,100	49,350	

***Supplemental Table X*: **Genetic correlation of coronary artery disease and peripheral artery disease with six common risk factors and CAD with PAD.

RISK FACTOR	PMID	CORONARY ARTERY DISEASE GENETIC CORRELATION (95%CI)	*P*	PERIPHERAL ARTERY DISEASE GENETIC CORRELATION (95%CI)	*P*
BODY MASS INDEX	25673413	0.22 (0.16,0.28)	9.8×10^{-12}	0.26 (0.13,0.40)	9.4×10^{-5}
CORONARY ARTERY DISEASE	26343387	-	-	0.58 (0.44,0.71)	1.1×10^{-16}
DIABETES (TYPE 2)	22885922	0.37 (0.27,0.47)	7.7×10^{-13}	0.38 (0.19,0.58)	1.0×10^{-4}
HDL CHOLESTEROL	24097068	-0.28 (-0.20,-0.36)	2.1×10^{-11}	-0.18 (-0.07,-0.30)	1.8×10^{-3}
LDL CHOLESTEROL	24097068	0.22 (0.12,0.32)	2.4×10^{-5}	0.13 (0.02,0.23)	0.02
TRIGLYCERIDES	24097068	0.27 (0.19,0.35)	8.1×10^{-12}	0.15 (0.04,0.27)	9.5×10^{-3}
SYSTOLIC BLOOD PRESSURE	UKB automated reading	0.34 (0.28,0.40)	4.7×10^{-29}	0.36 (0.24,0.48)	2.4×10^{-9}

Supplemental Table XI: Members of the SUMMIT consortium

Partner	Name	Position
1	**Michael Mark**	**Coordinator,WP6 leader**
Boehringer-Ingelheim	Markus Albertini	Project manager
Ingelheim,Germany	Carine Boustany	Chronic Kidney Disease,Head of Lab
	Alexander Ehlgen	Transmed
	Martin Gerl	Biomarker & Bioanlysis,Groupleader
	Jochen Huber	In vivo Scientist CMDR,Head of Lab
	Corinna Schölch	Biomarker & Bioanlysis,Head of Lab
	Heike Zimdahl-Gelling	Pharmacogenomics,Head of Lab
2	**Leif Groop**	Prof. Endocrinology; Coordinator Managing entity IMI-JU; PI; **WP1 and WP6 leader**
Lund University	Elisabet Agardh	Prof. Ophthalmology
Clinical Research Centre	Emma Ahlqvist	Postdoc
Malmö,Sweden	Tord Ajanki	Communication strategist
	Nibal Al Maghrabi	Research nurse
	Peter Almgren	Biostatistician
	Jan Apelqvist	Diabetologist
	Eva Bengtsson	Assis. Prof. Cardiovascular research
	Lisa Berglund	Postdoc
	Harry Björckbacka	Assis. Prof. Cardiovascular research
	Ulrika Blom-Nilsson	LUDC administrator
	Mattias Borell	Website,server management
	Agneta Burström	Research nurse
	Corrado Cilio	Assoc. Prof. Cellular autoimmunity
	Magnus Cinthio	Assist. Prof. Electrical Measurements,Lund Technical University
	Karl Dreja	Nephrologist
	Pontus Dunér	Postdoc Exp. Cardiovasc. Research
	Daniel Engelbertsen	PhD student Exp. Cardiovasc. Research
	Joao Fadista	Postdoc
	Maria Gomez	Assoc. Prof. Cardiovascular disease,**WP4 co-leader**
	Isabel Goncalves	Assis. Prof. Cardiovascular research
	Bo Hedblad	Prof. Cardiovascular epidemiology
	Anna Hultgårdh	Prof. Vessel Wall Biology
	Martin E. Johansson	Pathologist
	Cecilia Kennbäck	Laboratory Engineer
	Jasmina Kravic	Database manager
	Claes Ladenvall	Genetic statistician
	Åke Lernmark	Prof. Type 1 diabetes and celiac disease
	Eero Lindholm	Physician,Researcher Diabetic Complications

Partner	Name	Position
	Charlotte Ling	Assist. Prof. Epigenetics
	Holger Luthman	Prof. Medical genetics
	Olle Melander	Assoc. Prof. Hypertension and cardiovascular disease
	Malin Neptin	Biomedical analyst
	Jan Nilsson	Prof. Experimental Cardiovascular research,**WP3 leader**
	Peter Nilsson	Prof. Internal medicine
	Tobias Nilsson	PhD student Electrical Measurements,Lund Technical University
	Gunilla Nordin Fredriksson	Prof. Cardiovascular research
	Marju Orho-Melander	Prof. Genetic epidemiology
	Emilia Ottoson-Laakso	PhD student
	Annie Persson	Research nurse
	Margaretha Persson	Laboratory Engineer
	Mats-Åke Persson	Database manager
	Jacqueline Postma	Project manager
	Elisabeth Pranter	Research nurse
	Sara Rattik	PhD student Exp. Cardiovasc. Research
	Gunnar Sterner	Chief physician Internal Medicine Research Unit
	Lilian Tindberg	Research nurse
	Maria Wigren	Postdoc Exp. Cardiovasc. Research
	Anna Zetterqvist	PhD student
	Mikael Åkerlund	Postdoc
	Gerd Östling	Laboratory Engineer
3	**Timo Kanninen**	Technical director; PI
Biocomputing Platforms	Anni Ahonen-Bishopp	Software development manager
(BC Platforms)	Anita Eliasson	Financial and administrative director
Espoo,Finland	Timo Herrala	System (server) specialist
	Päivi Tikka-Kleemola	Service manager
4	**Anders Hamsten**	Prof. Cardiovascular disease; Atherosclerosis Research Unit; PI
Karolinska Institute	Christer Betsholtz	Prof. Vascular biology
Stockholm,Sweden	Ami Björkholm	Administrator
	Ulf de Faire	Professor emeritus Cardiovascular epidemiology
	Fariba Foroogh	Research engineer

Partner	Name	Position
	Guillem Genové	Scientist
	Karl Gertow	Research Assist. Prof. Cardiovascular genetics
	Bruna Gigante	Assoc. Professor Cardiovascular epidemiology
	Bing He	Postdoc
	Karin Leander	Assoc. Professor Cardiovascular epidemiology
	Olga McLeod	Postdoc
	Maria Nastase-Mannila	Postdoc
	Jaako Patrakka	Postdoc
	Angela Silveira	Assoc. Prof. Cardiovascular genetics
	Rona Strawbridge	Postdoc
	Karl Tryggvason	Prof. Medical Chemistry
	Max Vikström	Statistician
	John Öhrvik	Professor
	Anne-May Österholm	Postdoc
5	**Barbara Thorand**	Nutritional scientist,epidemiologist
Helmholtz Centre	Christian Gieger	Statistician
Munich,Germany	Harald Grallert	Biologist
	Tonia Ludwig	Statistician
	Barbara Nitz	Scientist
	Andrea Schneider	Data manager
	Rui Wang-Sattler	Scientist
	Astrid Zierer	Statistician
6	**Giuseppe Remuzzi**	Institute director; PI
Mario Negri Institute for	Ariela Benigni	Head of department Molecular Medicine
Pharmacological Research	Roberta Donadelli	Scientist
	Maria Domenica Lesti	Researcher
Bergamo,Italy	Marina Noris	Head Laboratory Immunology and genetics of transplantation and rare diseases
	Norberto Perico	Senior scientist
	Annalisa Perna	Biostatistician
	Rossella Piras	Postdoc
	Piero Ruggenenti	Head of department Renal medicine,Assist. Prof. Nephrology and dialysis
	Erica Rurali	Postdoc
7	**David Dunger (att: Jane Horsford)**	Prof. Paediatrics; PI
University of Cambridge	Ludo Chassin	Senior Data Manager

Partner	Name	Position
UK	Neil Dalton,London	Clinical biochemistry
	John Deanfield,London	Paediatric cardiology
	Jane Horsford	PA to Prof. Dunger
	Clare Rice	Operations manager/financial contact
	James Rudd	Cardiovascular imaging
	Neil Walker	Head Data services
	Karen Whitehead	Technician
	Max Wong	Postdoc
8	**Helen Colhoun**	Prof. Public health and epidemiology; PI; Vice coordinator Managing entity; **WP2 leader**
	Fiona Adams	
University of Dundee	Tahira Akbar	PA to Helen Colhoun
Scotland	Jill Belch	Prof. Vasucular disease
	Harshal Deshmukh	PhD student
	Fiona Dove	
	Angela Ellingford	NHS Tayside Diabetic Retinopathy Screening Programme manager
	Bassam Farran	Statistician
	Mike Ferguson	Dean of research Biological chemistry and drug discovery
	Gary Henderson	
	Graeme Houston	Consultant radiologist/senior lecturer
	Faisel Khan	Reader,Vascular & Inflammatory Diseases Research Unit
	Graham Leese	Consultant diabetologist/reader
	Yiyuan Liu	PhD student
	Shona Livingstone	Senior statistician
	Helen Looker	Epidemiologist
	Margaret McCann	Project assistant
	Stuart McGurnaghan	Lead data programmer
	Andrew Morris	Prof. Diabetic medicine
	David Newton	
	Colin Palmer	Prof. Pharmacogenomics
	Ewan Pearson	Consultant diabetologist/senior lecturer
	Gillian Reekie	Research Nurse
	Natalie Smith	Research Nurse
9	**Angela Shore**	Prof. Cardiovascular Science,PI
Peninsula Medical School	Kuni Aizawa	Postdoc
Exeter,UK	Claire Ball	Research nurse

Partner	Name	Position
	Nick Bellenger	Cardiologist
	Francesco Casanova	Associate Research Fellow Vascular medicine
	Tim Frayling	Prof. Genetics
	Phil Gates	Senior lecturer Cardiovascular science
	Kim Gooding	Postdoc Vascular medicine
	Andrew Hatttersley	Prof. Molecular medicine
	Roland Ling	Consultant opthalmologist
	David Mawson	Research technician
	Robin Shandas	Prof. Bioengineering (Colorado)
	David Strain	Stroke physician,clinical lecturer
	Clare Thorn	Postdoc Vascular medicine
10	**Ulf Smith**	Prof. ; PI
University of Gothenburg	Ann Hammarstedt	Researcher Molecular and clinical medicine
Sweden	Hans Häring	Prof. University of Tübingen
	Oluf Pedersen	Prof. Steno Centre,Copenhagen
	Georgio Sesti	Prof. Universtiy of Catanzaro
11	**Per-Henrik Groop**	Prof. Diabetes genetics; PI
	Emma Fagerholm	PhD student,genetics
Folkhälsan	Carol Forsblom	Clinical coordinator
Helsinki,Finland	Valma Harjutsalo	
	Maikki Parkkonen	Laboratory manager
	Niina Sandholm	DSc(PhD); GWAS and bioinformatics
	Nina Tolonen	MD PhD
	Iiro Toppila	BSc,bioinformatician
	Erkka Valo	MSc,bioinformatician
12	**Veikko Salomaa**	Prof. Epidemiology; PI; **deputy leader WP2**
The National Institute for Health and Welfare	Aki Havulinna	DSc. (tech),statistician
Helsinki,Finland	Kati Kristiansson	Postdoc
	Pia Okamo	THL press officer
	Tomi Peltola	
	Markus Perola	Professor
	Arto Pietilä	Statistician
	Samuli Ripatti	Professor,Statistics
	Marketta Taimi	Research assistant
13	**Seppo Ylä-Herttuala**	Prof.; PI; **WP4 leader**
University of Eastern Finland	Mohan Babu	PhD student

Partner	Name	Position
Kuopio,Finland	Marike Dijkstra	PhD student
	Erika Gurzeler	PhD student
	Jenni Huusko	PhD student
	Ivana Kholová	Postdoc
	Markku Laakso	Prof.
	Mari Merentie	PhD student
	Marja Poikolainen	PA Prof Ylä-Herttuala
14	**Mark McCarthy**	Prof. Human type 2 diabetes; Oxford Centre for Diabetes,Endocrinology and Metabolism; Wellcome Trust Centre for Human Genetics; PI; **deputy leader WP1**
University of Oxford	Chris Groves	Technical staff
UK	Thorhildur Juliusdottir	PhD student
	Fredrik Karpe	PI OCDEM
	Vasiliki Lagou	Postdoc
	Andrew Morris	Wellcome Trust Senior Fellow; Bioinformatics and statistical genetics
	Will Rayner	Database manager
	Neil Robertson	Informatics
	Natalie van Zuydam	Postdoc
15	**Claudio Cobelli**	Prof. ; PI; **WP5 leader**
University of Padova	Barbara Di Camillo	Assist. Prof.
Italy	Francesca Finotello	PhD student
-	Francesco Sambo	Postdoctoral fellow
-	Gianna Toffolo	Prof.
-	Emanuele Trifoglio	PhD student
-	-	-
16	**Riccardo Bellazzi**	Prof. Bioengineering; PI; **deputy leader WP5**
	Nicola Barbarini	Postdoctoral fellow
University of Pavia	Mauro Bucalo	Software engineer
Italy	Christiana Larizza	Assist. Prof.
	Paolo Magni	Assoc. Prof.
	Alberto Malovini	Postdoctoral fellow
	Simone Marini	Postdoctoral fellow
	Francesca Mulas	Postdoctoral fellow
	Silvana Quaglini	Prof.
	Lucia Sacchi	Assist. Prof.
	Francesca Vitali	

Partner	Name	Position
17	**Ele Ferrannini**	Prof. Medicine; PI
	Beatrice Boldrini	Postdoctoral fellow
University of Pisa	Michaela Kozakova	Senior investigator Medical Pathophysiology
Italy	Andrea Mari	Senior researcher Biomedical engineering (ISIB-CNR,Padova)
	Carmela Morizzo	Biologist,Sonographer Cardiovascular ultrasound
	Lucrecia Mota	EGIR administrative office
	Andrea Natali	Assoc. Prof. Medicine
	Carlo Palombo	Assoc. Prof. Medicine; **deputy leader WP3**
	Elena Venturi	Researcher
	Mark Walker	Prof. Molecular diabetic medicine (Univ Newcastle-upon-Tyne)
18	**Carlo Patrono**	Prof.Pharmacology; PI
Catholic University of Rome	Francesca Pagliaccia	PhD student
Italy	Bianca Rocca	Assist. Prof. Pharmacology
19	**Pirjo Nuutila**	Prof. ; PI
University of Turku	Johanna Haukkala	PhD student
Finland	Juhani Knuuti	Prof. ; Director Turku PET Centre
	Anne Roivainen	Prof.
	Antti Saraste	Adj. Prof.
20	**Paul McKeague**	Prof. Genetic Epidemiology; PI
University of Edinburgh	Norma Brown	Research administrator,Public Health Services
Scotland	Marco Colombo	Bioinformaticist
21	**Birgit Steckel-Hamann**	Deputy coordinator; PI,Manager IMI,LRL
Eli Lilly	Krister Bokvist	Biostatistician
	Sudha Shankar	Diabetologist
	Melissa Thomas	Translational Science
22	**Li-ming Gan**	Prof.; Translational Science Director Cardiovascular Disease; PI,**WP3 leader**
AstraZeneca	Suvi Heinonen	PhD,Internal AZ postdoc,Bioscience
	Ann-Cathrine Jönsson-Rylander	PhD,Assoc. Prof.,Team Leader Bioscience,**WP4 leader**
	Remi Momo	Postdoctoral fellow
	Volker Schnecke	Informatician Translational Science,**WP5 leader**

Partner	Name	Position
	Robert Unwin	Translational Science Director Diabetic Nephropathy
	Anna Walentinsson	Geneticist Translational Science
	Carl Whatling	Bioscientist
23	**Everson Nogoceke**	Pre-clinical and clinical aspects of metabolic and vascular disease; PI; **WP2 leader**
Roche	Gonzalo Durán Pacheco	Senior Research Statistician
	Ivan Formentini	Biomarker & Experimental Medicine Leader
	Thomas Schindler	Pre-clinical and clinical and clinical biomarkers
24	**Piero Tortoli**	Professor of Electronics
University of Florence	Luca Bassi	Postdoctoral fellow
	Enrico Boni	Postdoctoral fellow
	Alessandro Dallai	Postdoctoral fellow
	Francesco Guidi	Technician
	Matteo Lenge	PhD student
	Riccardo Matera	PhD student
	Alessandro Ramalli	PhD student
	Stefano Ricci	Assist. Prof.
	Jacopo Viti	PhD student
25	**Bernd Jablonka**	SAD internal IMI coordinator
Sanofi-aventis	Dan Crowther	Biomarker researcher
	Johan Gassenhuber	Biostatistician
	Sibylle Hess	Biomarker researcher
	Thomas Hübschle	Pharmacologist Diabetes
	Hans-Paul Juretschke	Imaging
	Hartmut Rütten	Head Translational Medicine
	Thorsten Sadowski	Pharmacologist Diabetes
	Paulus Wohlfart	Pharmacologist Diabetes
26	**Julia Brosnan**	Biochemist,(pre)clinical research CVD,Pfizer US; **WP2 leader**
Pfizer	Valerie Clerin	Cardio-renal biologist,WP2
	Eric Fauman	Computational biologist
	Craig Hyde	Statistician
	Anders Malarstig	Human genetics,Pfizer Europé; **WP1 leader**
	Nick Pullen	Renal Disease Research Director
	Mera Tilley	
	Theresa Tuthill	Imaging specialist

Partner	Name	Position
	Ciara Vangjeli	Cardiovascular genetic epidemiologist,Pfizer Europe
	Daniel Ziemek	Computational biologist

Supplemental Table XII: Members of the GoLEAD consortium

**Study/Partner**	**Name**	**Institution**
WESDR	Barbara Klein	University of Wisconsin-Madison
Corogene	Veikko Salomaa	University of Helsinki
SDR	Emma Ahlqvist	University of Lund
	Leif Groop	University of Helsinki
	Eero Lindholm	University of Lund
FinnDiane	Per-Henrik Groop	University of Helsinki
	Emma Dalhström	University of Helsinki
	Carol Forsblom	University of Helsinki
	Niina Sandholm	University of Helsinki
DCCT/EDIC	Andrew Paterson	University of Toronto
GoDARTS	Colin NA Palmer	University of Dundee
	Helen M Colhoun	University of Edinburgh
	Natalie R van Zuydam	University of Oxford
HPS	Jemma C Hopewell	University of Oxford
	Alex Stiby	University of Oxford
deCODE	Sólveig Grétarsdóttir	deCODE
	Guðmar Þorleifsson	deCODE
	Unnur þorsteinsdóttir	deCODE
	Kari Stefansson	deCODE
Mayo Clinic	Iftikhar Kullo	Mayo Clinic Rochester
	Mariza de Andrade	Mayo Clinic Rochester
UKBB	Mark I McCarthy	University of Oxford
	Anubha Mahajan	University of Oxford
	Natalie R van Zuydam	University of Oxford
University of Liverpool	Andrew P Morris	University of Liverpool
EAS	Jackie Price	University of Edinburgh

Supplemental Figures

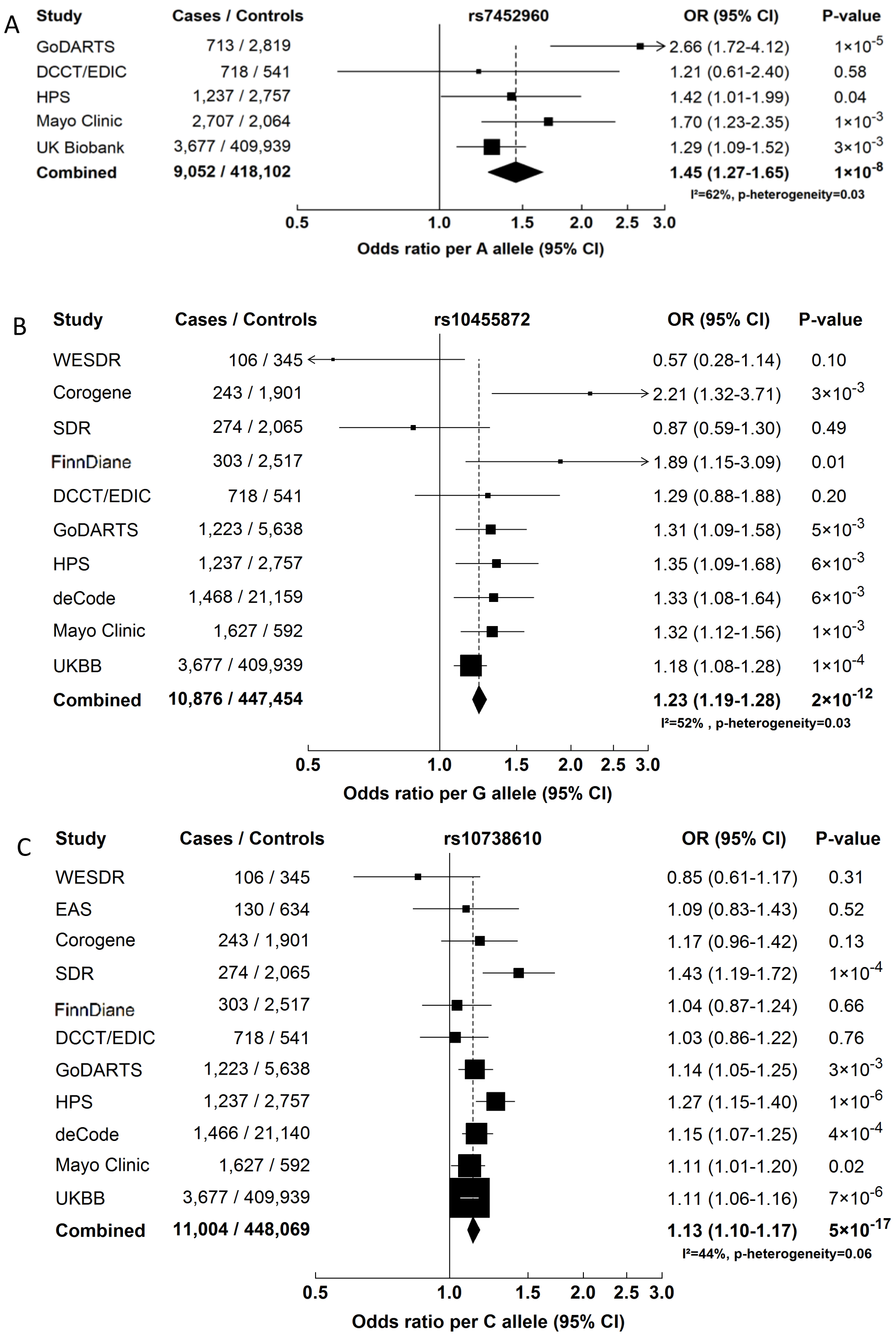

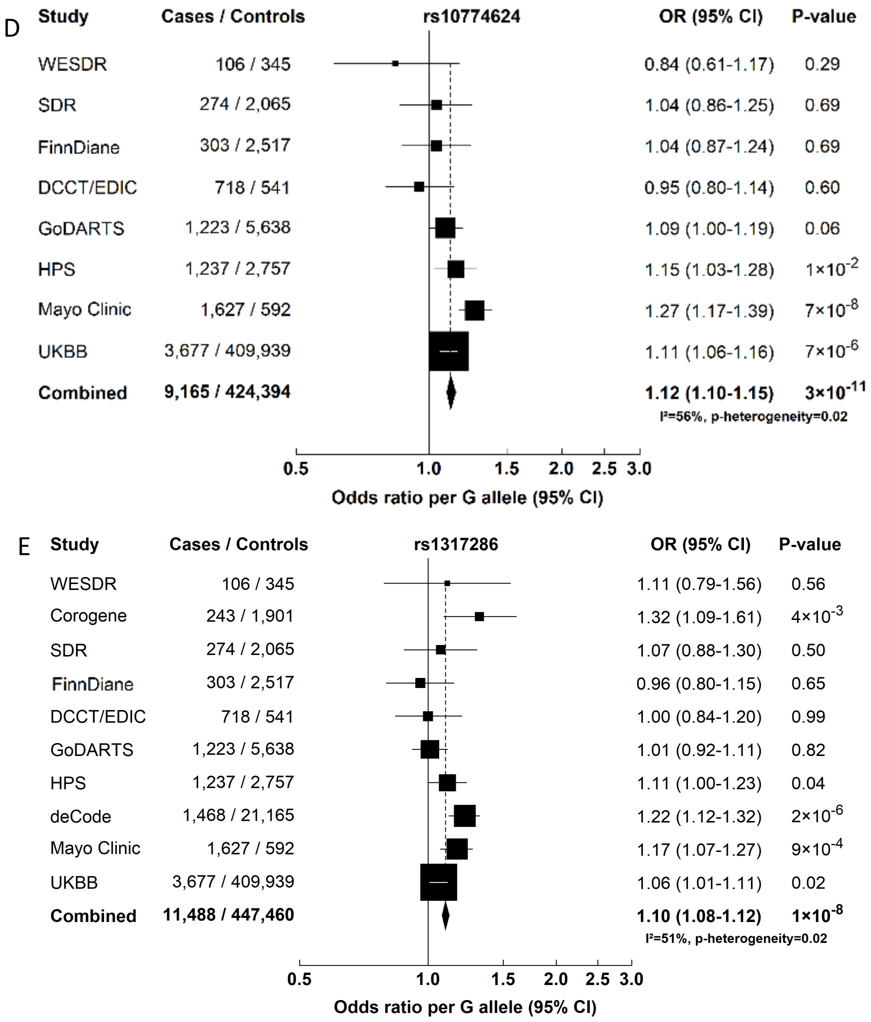

Supplemental Figure I: Forest plots of the lead variants for genome-wide significant loci ($p \leq 5 \times 10^{-8}$) in the combined analysis of peripheral vascular disease. A) *Lipoprotein A*; B) *Lipoprotein A*; C) *CDKN2A-BAS1; D) PTPN11; and E) Cholinergic Receptor Nicotinic Alpha 5 Subunit*

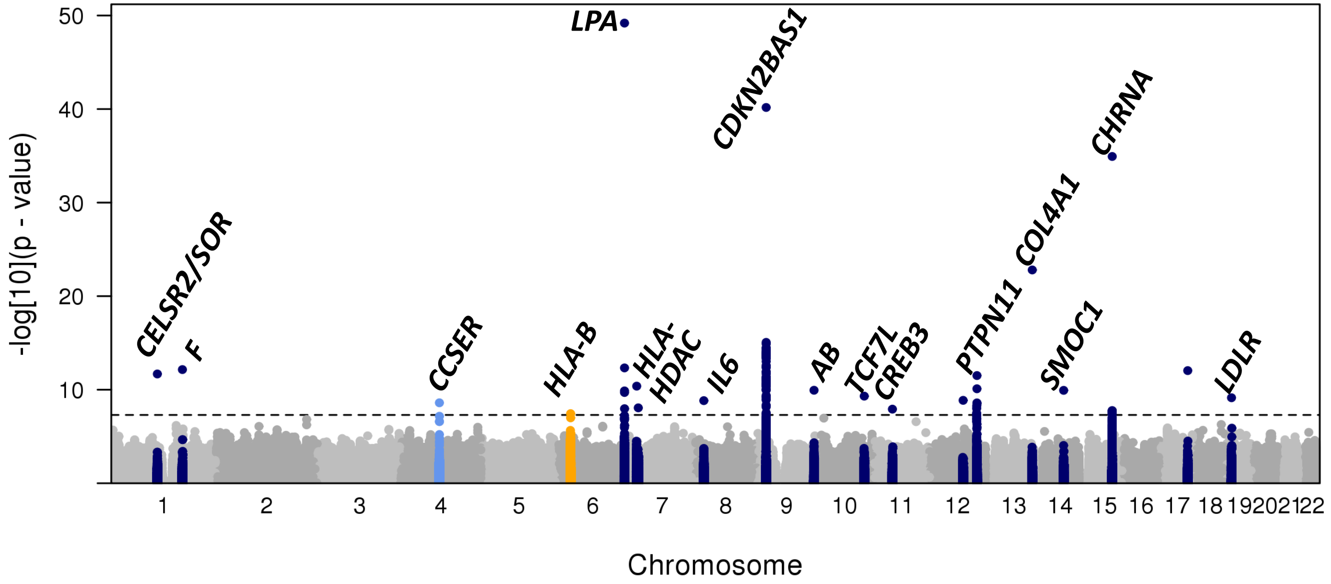

Supplemental Figure II: A Manhattan plot of results from all analyses yielding genome-wide significant associations. Dark blue indicates the p value estimated from combining allele effects across GoLEAD, Million veterans program and the UK Biobank for published lead variants, and underneath these are the p values estimated in GoLEAD and UKBB for variants within 1Mb of the reported lead variant. Light blue indicates variants within 1Mb of a variant associated with PAD in individuals with diabetes, and orange for variants associated with PAD in ever smokers.

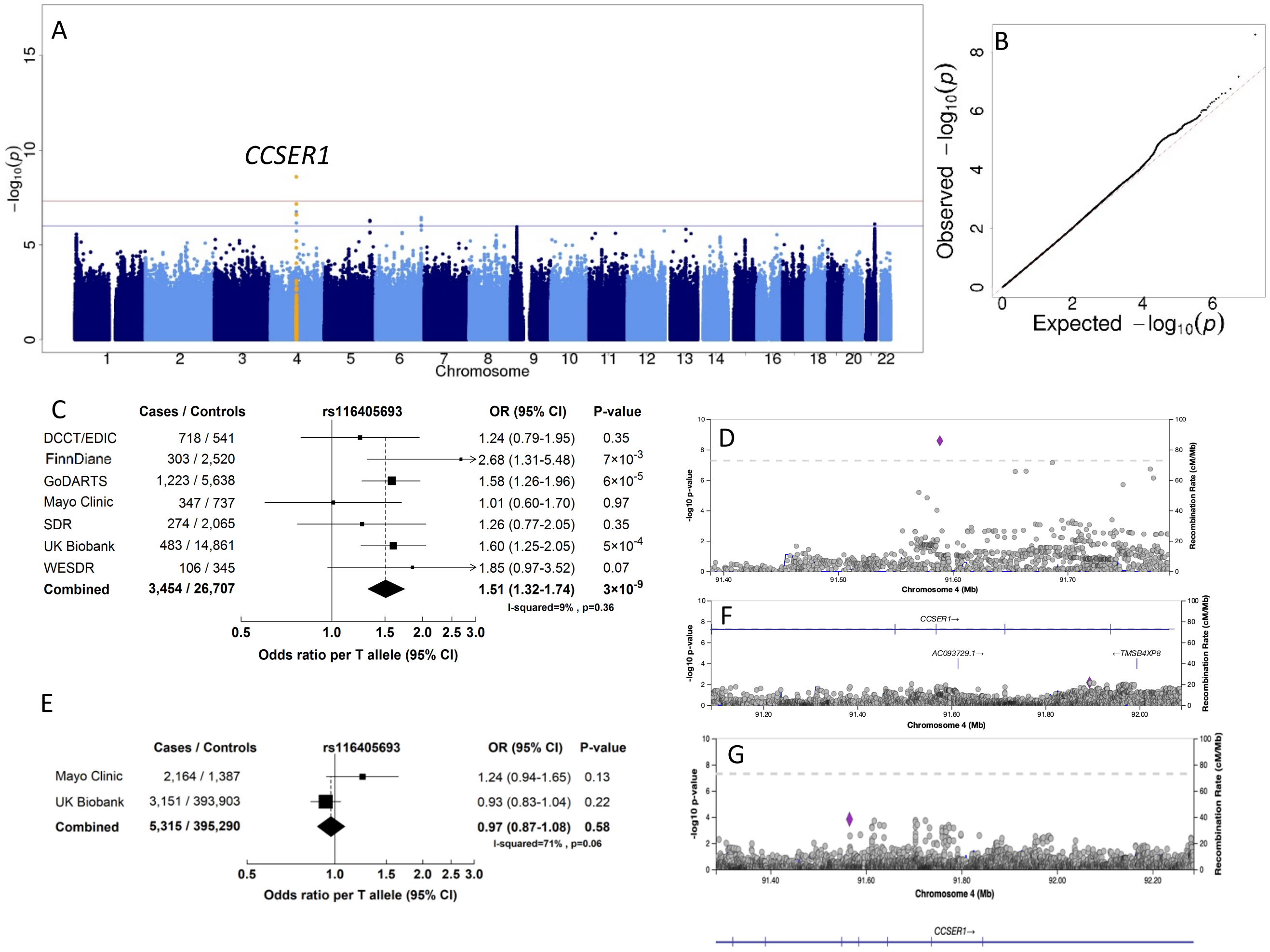

Supplemental Figure III: A) Manhattan and B) QQ-plot of association *p* value for peripheral artery disease in individuals with diabetes. C) A forest plot of allelic effects by study for rs116405693 near *CCSER1* in subjects with diabetes and D) Locuszoom plot of the *CCSER1* region in subjects with diabetes. E) Forest plot of rs116405693 in subjects without diabetes, F) a Locuszoom plot of the *CCSER1* region in subjects without diabetes and G) a Locuszoom plot fo the *CCSER1* region from the primary PAD meta-analysis.

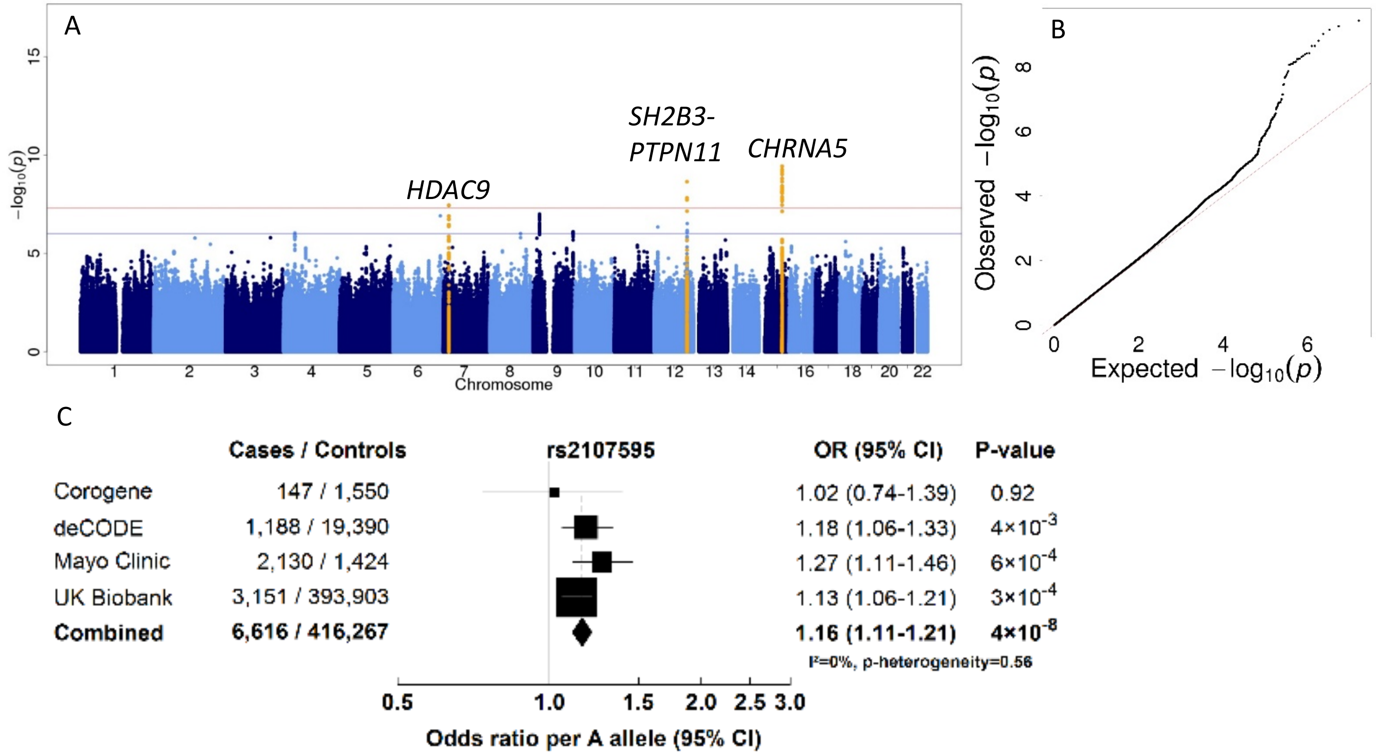

Supplemental Figure IV: A) Manhattan and B) QQ-plot of association p value for peripheral artery disease in individuals without diabetes. C) A forest plot of allelic effects by study for rs2107595 near *HDAC9*.

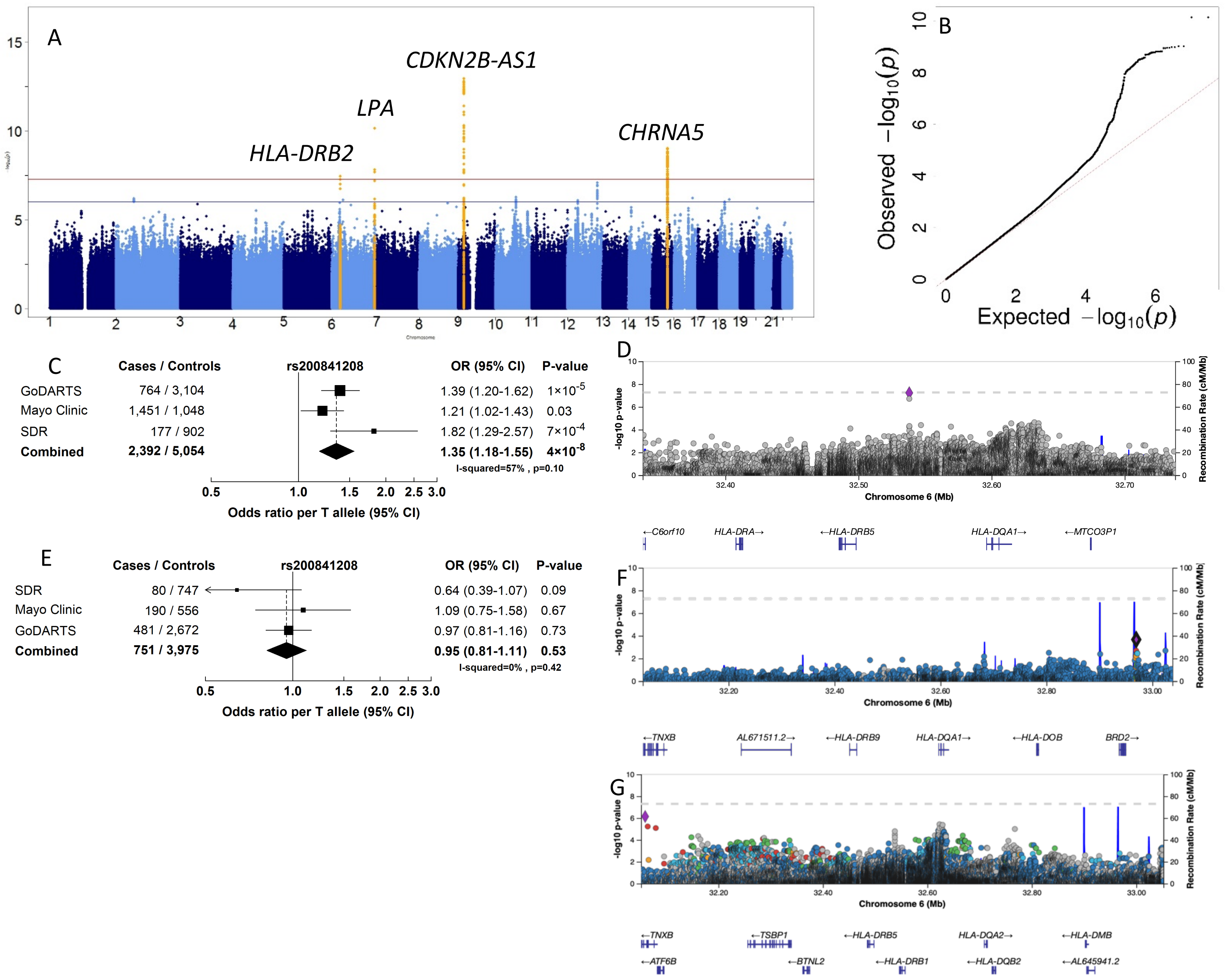

Supplemental Figure V: A) Manhattan and B) QQ-plot of association *p* value for peripheral artery disease in ever smokers. C) A forest plot of allelic effects by study for rs200841208 in ever smokers and in E) A forest plot of rs200841208 in never smokers; F) A Locuszoom plot of in never smokers near *HLA-DRB1* and G) a Locuszoom plot of the region from the primary PAD meta-analysis.

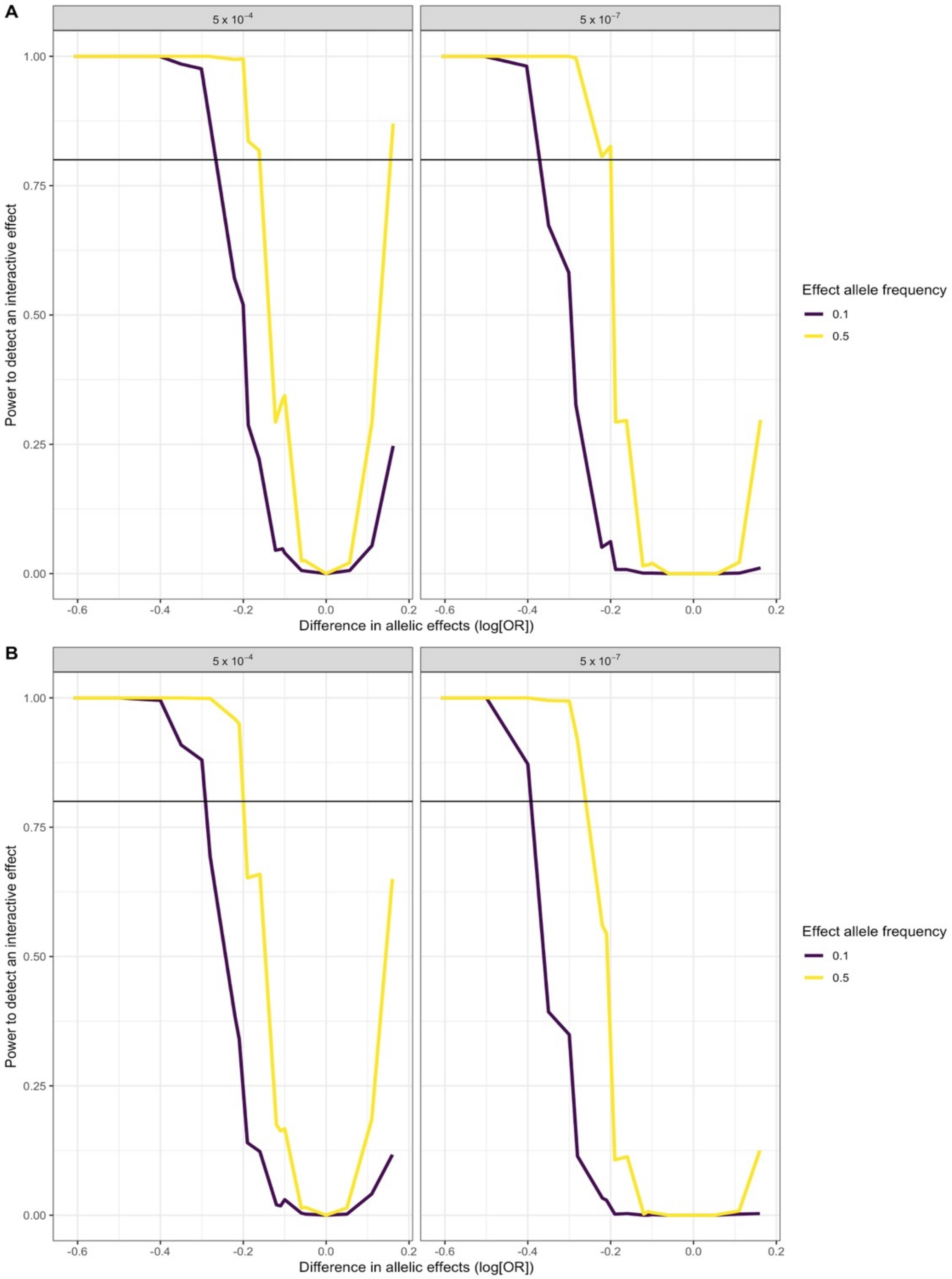

Supplemental Figure VI: Post hoc power analysis to detect interactive effects in: A) smokers (N_{cases}=7,404 and $N_{controls}$=205,693) vs non-smokers (N_{cases}=2,414 and $N_{controls}$=239,806); and B) diabetes (N_{cases}=3,846 and $N_{controls}$=28,881) vs no diabetes.
